# Carbon source acts as a deterministic filter shaping microbial succession and rare-abundant decoupling in soil bacterial communities

**DOI:** 10.1093/ismeco/ycag108

**Published:** 2026-04-20

**Authors:** Leonardo Stari, Hiromi Kato, Kouhei Kishida, Yoshiyuki Ohtsubo, Michio Kondoh, Yuji Nagata

**Affiliations:** Graduate School of Life Sciences, Tohoku University, Katahira 2-1-1, Aoba-ku, Sendai 980-8577, Japan; Graduate School of Life Sciences, Tohoku University, Katahira 2-1-1, Aoba-ku, Sendai 980-8577, Japan; Graduate School of Life Sciences, Tohoku University, Katahira 2-1-1, Aoba-ku, Sendai 980-8577, Japan; Graduate School of Life Sciences, Tohoku University, Katahira 2-1-1, Aoba-ku, Sendai 980-8577, Japan; Graduate School of Life Sciences, Tohoku University, Katahira 2-1-1, Aoba-ku, Sendai 980-8577, Japan; Graduate School of Life Sciences, Tohoku University, Katahira 2-1-1, Aoba-ku, Sendai 980-8577, Japan

**Keywords:** bacterial community, taxonomic structure, community succession, 16S rRNA gene amplicon sequencing, deterministic selection, rare biosphere

## Abstract

The principles governing microbial community assembly and the interplay between deterministic selection and stochasticity remain central debates in ecology. We investigated how chemically diverse carbon sources act as ecological filters, shaping soil bacterial communities. Using replicated microcosms amended with distinct substrates (glucose, succinate, naphthalene, phenanthrene, or γ-hexachlorocyclohexane (γ-HCH)) or under starvation, we tracked community trajectories via high-frequency 16S ribosomal RNA (rRNA) gene sequencing (29 timepoints, triplicate) and quantitative ecological modeling. Null model analysis confirmed the carbon source as the primary deterministic filter, enforcing high reproducibility (homogeneous selection governing ~74% of assembly among replicates) and overriding stochastic effects. Crucially, abundant (>1%) and rare (<0.1%) taxa exhibited decoupled assembly mechanisms. While abundant taxa were driven largely by dispersal limitation (~59%) and variable selection, the rare biosphere displayed a temporal regime shift. Unlike the immediate response of dominant taxa, rare taxa transitioned from stochastic isolation to strong deterministic selection (surging to ~50%) only during later successional stages. This reframes the rare biosphere as a “latent responder” reservoir recruited by metabolic byproducts rather than the primary substrate. Additionally, time-resolved interaction networks revealed that under severe stress from toxicity or starvation, interactions shifted from competitive exclusion to facilitation (e.g. necromass scavenging). These patterns provide strong empirical support for the Stress Gradient Hypothesis in a microbial context. Collectively, our findings demonstrate how deterministic filtering and stress-mediated cooperation jointly structure ecosystems, providing a high-resolution temporal dataset to further interrogate these fundamental ecological principles.

## Graphical Abstract

**Graphical Abstract:**
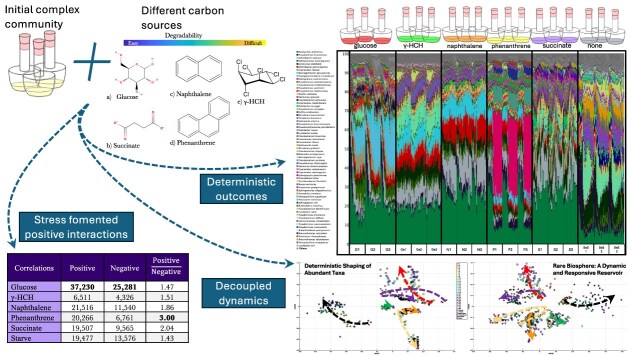

## Introduction

Microbial communities are essential drivers of ecosystem functions, yet the processes governing their assembly are complex and not fully elucidated. A critical factor is the availability and nature of carbon sources, which can steer the successional trajectory of bacterial populations [1–3]. Historically, bacterial succession was viewed as the temporal evolution of community structure driven by environmental constraints [4]. While recent work has challenged the universality of these patterns [5], classifying succession based on carbon inputs remains crucial [3]. Previous research has explored these dynamics using ecological models [6], examining the roles of competition [7] community resilience [8], and emergent properties [9]. However, such models are often limited by a lack of high-resolution, empirical time-series data from complex communities under controlled selective pressures. Generating these foundational datasets is therefore essential for moving beyond theoretical predictions to a mechanistic understanding of community assembly.

A key challenge in this understanding is the “rare biosphere,” a vast array of low-abundance taxa [10], thought to act as a seed bank of genetic diversity that can rapidly proliferate when environmental parameters shift [11, 12]. Despite recognition as integral to ecosystem function, it remains an open question whether they are regulated by the same ecological forces. Clarifying this knowledge gap is essential for understanding microbial resilience and for testing hypotheses about community assembly under changing environments [13, 14]. Recent evidence suggests a dichotomy: abundant taxa are shaped by deterministic selection, while the rare subcommunity appears driven by stochastic drift [15]. However, we question whether this rare biosphere is truly stochastic or if it functions as a “latent” reservoir responding deterministically to delayed cues after abundant members modify the environment.

In laboratory microcosms, microbial succession is guided by a combination of deterministic environmental pressures and stochastic events [16, 17]. While the link between biodiversity and ecosystem function is strong in controlled settings, it is less consistent in natural ecosystems [18]. Our research addresses this by focusing on the interactions within a soil-derived bacterial community and how its population structure evolves under different substrate conditions.

In this study, we explore these dynamics by tracking a soil bacterial population exposed to carbon sources with diverse biodegradability: glucose, succinate, naphthalene, phenanthrene, and the persistent pesticide γ-HCH. Using high-frequency sampling (29 timepoints over 14 days), we tested three hypotheses: (i) the specific carbon source acts as a powerful deterministic filter overriding stochasticity; (ii) dominant and rare members exhibit decoupled successional dynamics reflecting distinct ecological strategies; and (iii) interspecies interactions shift from competition to facilitation under stress, as predicted by the Stress Gradient Hypothesis. This dense V3–V4 amplicon sequencing dataset, which will be made publicly available, provides a robust foundation for the detailed analyses that follow and for future meta-analyses of microbial succession.

## Materials and methods

### Experimental design and cultivation

All chemicals and reagents were obtained from Wako Pure Chemical Industries, Ltd. (Osaka, Japan) unless specifically stated.

A bacterial community was sourced from soil from Ehime Prefecture, Japan [19]. A preculture was established by inoculating 1/10 strength W medium supplemented with 1.5% (w/v) soil extract (8.32 mM carbon equivalent). Soil extract was included in the medium, not as an inoculum source but to supplement the minimal medium with trace micronutrients and dissolved organic matter native to the sampling site. This was done to minimize “bottle effects” and stochastic extinction of auxotrophic rare taxa during the acclimation phase. The preculture was incubated until the absorbance stabilized, ensuring a consistent starting community for all experiments.

The main experiment was conducted in biological triplicates. Each replicate consisted of a 500 ml Sakaguchi flask containing 200 ml of 1/10X W medium, maintained at 30°C with agitation at 130 RPM. This temperature was selected to accelerate metabolic rates and successional dynamics to fit within the high-resolution 14-day observation window.

**Figure 1:**
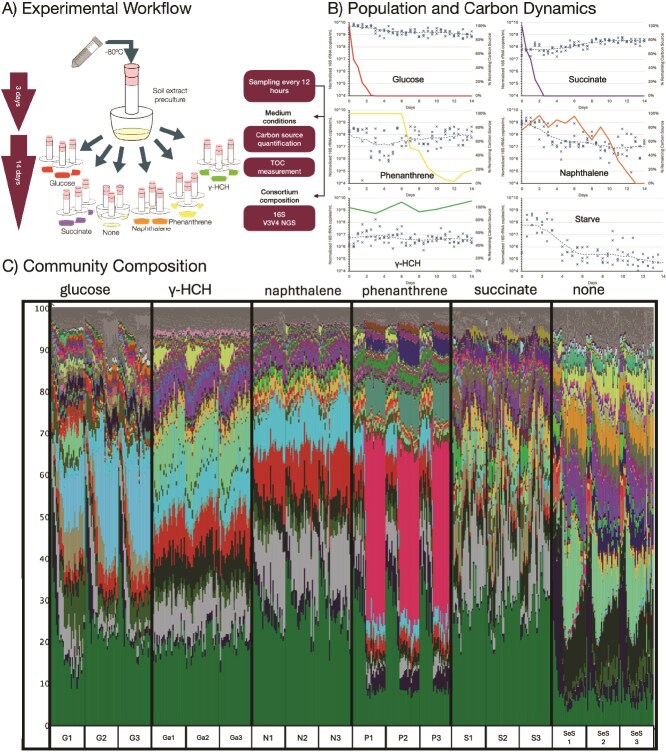
Experimental design and the effect of carbon source on bacterial growth, substrate consumption, and community composition. (A) Experimental design. A soil-derived bacterial community was inoculated into microcosms containing 1/10X W medium supplemented with soil extract until stable (around 3 days) after which it was separated into Sakaguchi flasks with one of five distinct carbon sources (glucose, succinate, naphthalene, phenanthrene, or γ-HCH) or no added carbon (starved control). The experiment was conducted in biological triplicates, and samples were collected every 12 h over 14 days for analysis of carbon source concentration, total organic carbon (TOC), and bacterial community structure via 16S rRNA gene amplicon sequencing. (B) Substrate consumption and bacterial growth. Temporal dynamics for each treatment are shown. Individual data points (“x” markers) and rolling window average (window size 5, dashed line) illustrate the change in total bacterial abundance (normalized 16S rRNA gene copies/ml, left *y*-axis), representing the distribution of three biological triplicates at each time point. The solid line corresponds to the percentage of the initial carbon source remaining in the culture (right *y*-axis). Rapid consumption of easily metabolized substrates like glucose and succinate supported the highest population densities, while the recalcitrant pesticide γ-HCH was not degraded and, like starvation, led to a decline in population. (C) Community composition. A stacked bar chart shows the temporal changes in the relative abundance of the top 65 OTUs with the rest grouped as others (gray). Each column represents a single time point, with time progressing from left to right within each treatment block (e.g. G1, G2, G3 for glucose triplicates; N1–3 for naphthalene). The distinct and highly reproducible taxonomic patterns observed for each carbon source demonstrate that environmental selection is the dominant force shaping community structure. For clarity, the detailed taxonomic legend is omitted here; please refer to Supplementary Fig. S1 for the full taxonomic key and expanded charts.

Microcosms were cultivated in sterilized Sakaguchi flasks sealed with breathable sterile silicone plugs to allow gas exchange while preventing airborne contamination. Sampling was performed twice daily for 14 days (see Fig. 1); initial time point (T0) samples were collected ~1 h after substrate addition. All sampling procedures were conducted within a laminar flow hood using sterile serological pipettes and tips to maintain aseptic conditions. Sampling was performed by aseptically removing 1 ml of culture fluid at each time point. No volume replacement was performed to avoid altering nutrient concentrations or diluting metabolic byproducts mid-experiment; the total volume reduction over the 14-day period was <15% of the initial volume.

The medium was supplemented with one of five carbon sources at a 50 mM carbon equivalent concentration: glucose (1.5 g/l), disodium succinate (2.027 g/l), naphthalene (0.64 g/l), phenanthrene (0.63 g/l), or γ-HCH (2.42 g/l). A “starved” control with no added carbon source was also included.

The 1/10X W medium consisted of 10 ml 10X W medium and 10 ml of 100X W salt solution per liter [20]. The soil extract was prepared by suspending 400 g of soil in 1 l of distilled water in a 2 or 3 l Erlenmeyer flask, autoclaving at 121°C for 30 min, and then filtering using a vacuum filtration flask. The filtered extract was dispensed into several medium bottles and autoclaved again at 121°C for 20 min.

### Chemical analysis

Carbon source concentrations were measured using specific analytical methods. Glucose and succinate were quantified using colorimetric assay kits (Dojindo and Sigma-Aldrich, respectively).

Naphthalene and phenanthrene measurements were conducted using an Agilent 7890A gas chromatograph, connected to a 5975C mass spectrometer and an autosampler 7693A (Agilent Technologies, USA). A DB-1MS column (30 m × 0.25 mm i.d., film thickness: 0.25 μm, J & W Scientific) was used for the analysis. Oven temperature was held at 125°C for 4 min, then ramped up by 5°C per minute until reaching 210°C, and held at this temperature for 4 min; an injection port temperature of 250°C; a source temperature of 230°C; high purity (G1) helium as the carrier gas with a flow rate of 1 ml/min; and a mass spectrometer scan range of 10–500 amu.

γ-HCH content was analyzed by gas chromatography with an electron capture detector (GC-ECD; GC-17A; Shimadzu, Kyoto, Japan) and an Rtx-1 capillary column (30 m × 0.25 mm × 0.25 μm; Restek, Bellefonte, PA, USA), with samples prepared by following these steps: −100 μl of sample was extracted using 100 μl ethyl acetate with 2 mg/l Dieldrin (C_12_H_8_Cl_6_O) as an internal standard; the mixture was vortexed for 1 min and centrifuged at 15 000 RPM for 5 min at 4°C. Oven temperature was held at 160°C for 1 min, then ramped up by 4°C per minute until reaching 280°C, and held at this temperature for 3 min; the injector and detector temperatures were set at 260°C and 300°C, respectively; helium gas was used as the carrier gas with a flow rate of 1 ml/min.

Total organic carbon (TOC) was measured using a TOC analyzer (TOC-L, Shimadzu, Kyoto, Japan) with an automatic sampler (ASI-V, Shimadzu). One hundred microliters of the sample were diluted in 9 ml of deionized water and loaded into the autosampler. A 3-point standard curve with glucose was used for TOC measurement, while sodium carbonate was used for inorganic carbon (IC) determination.

### DNA extraction and population quantification

DNA was extracted from 500 μl sample pellets using a NucleoSpin Tissue kit (Macherey–Nagel) with a lysozyme treatment step. An artificial pEGFP plasmid (10^7^ copies/μl) was added as an internal standard for quantitative analysis.

Total bacterial abundance, used to track population dynamics, was determined by quantitative PCR (qPCR) of the 16S rRNA gene using universal primers 341F (5′-CCTACGGGAGGCAGCAG-3′) and 518R (5′-ATTACCGCGGCTGCTGG-3′) on a CFX Connect real-time system (Bio-Rad). The EGFP gene was quantified with primers 1070R and 960F to normalize for DNA extraction efficiency.

### 16S rRNA gene amplicon sequencing and Bioinformatic analysis

The V3–V4 hypervariable regions of the 16S rRNA gene were amplified following the Illumina 16S Metagenomic Sequencing Library Preparation protocol [3]. The first PCR step used the following full-length primers, which include Illumina adapter overhangs (standard text), heterogeneity spacers (N), and the target-specific sequences (bold): 341F (5'-ACACTCTTTCCCTACACGACGCTCTTCCGATCT-NNNNN-**CCTACGGGNGGCWGCAG**-3′) and 805R (5′-GTGACTGGAGTTCAGACGTGTGCTCTTCCGATCT-NNNNN-**GACTACHVGGGTATCTAATCC**-3′). The resulting amplicons were indexed in a second PCR step and sequenced on an Illumina MiSeq platform (paired-end).

The raw sequencing data were processed using a custom script using fastp [21] and vsearch [22] for merging, dereplication, and clustering (97% identity). Taxonomy assignment is performed employing vsearch, utilizing the EzBioCloud 16S Database [23]. To ensure the robustness of our ecological conclusions against clustering biases, a parallel zero-radius Operational Taxonomic Unit (zOTU) analysis was conducted (unoise3). This quantitative comparison confirmed that the global successional trajectories and subcommunity decoupling patterns were mathematically consistent regardless of the bioinformatic pipeline (Supplementary Table S1).

### Phylogenetic tree construction and ecological process analysis

To generate a robust phylogenetic framework for the 97% OTUs, representative sequences were placed into the precomputed SILVA 128 reference phylogeny using the SEPP fragment insertion algorithm (QIIME 2 plugin qiime fragment-insertion) [24]. This fragment-insertion approach onto a curated full-length 16S backbone was explicitly chosen over de novo tree construction (e.g. FastTree). Short V3–V4 amplicons lack the deep-branching phylogenetic signal required to accurately resolve ancestral nodes, and using a curated backbone prevents the introduction of severe topological artifacts during downstream null-model (βNTI) calculations. A pairwise patristic distance matrix was calculated using the resulting tree.

To partition the ecological processes driving community assembly (Selection vs. Dispersal vs. Drift), we employed the null model framework described by Stegen *et al*. [25]. Analyses were performed in MATLAB. First, we calculated the β-Nearest Taxon Index (βNTI) using relative abundance data. |βNTI| values >2 indicate dominance by deterministic selection (Homogeneous: <−2; Variable: > + 2). Second, for pairwise comparisons not driven by selection (|βNTI| < 2), we calculated the Raup-Crick metric (RC_bray) using a null model that simulates stochastic community assembly based on integer-rounded count data. RC_bray values > + 0.95 or <−0.95 indicate Dispersal Limitation or Homogenizing Dispersal, respectively, while values between −0.95 and + 0.95 indicate Undominated processes (Ecological Drift).

### Data and statistical analysis

**Figure 2:**
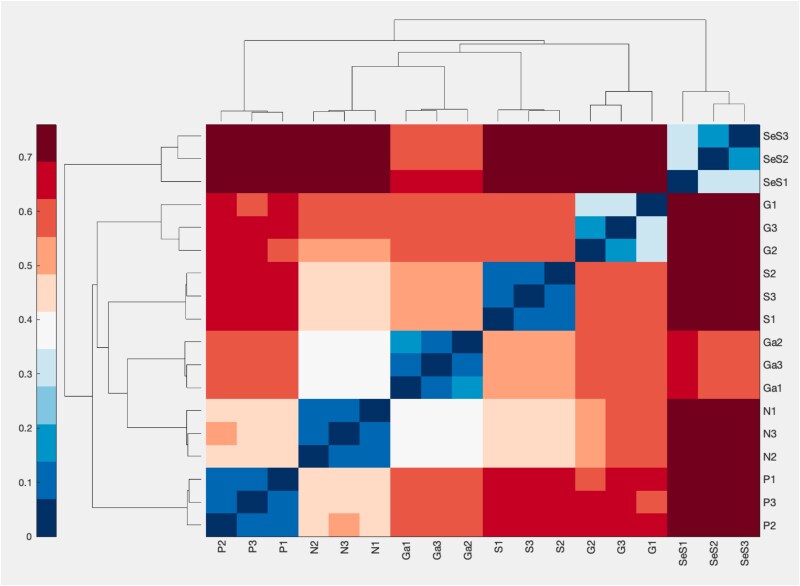
Bray–Curtis dissimilarity among biological triplicates. The clustergram shows low dissimilarity (dark blue) within each set of triplicates (e.g. G1, G2, G3 for glucose), indicating high reproducibility. The clustering reveals that communities from glucose and phenanthrene are distinct from each other and from the succinate/naphthalene/γ-HCH group.

All statistical analyses were performed in MATLAB with the addition of the Fathom Toolbox [26]. Prior to analysis, data were normalized using Total Sum Scaling (TSS) for standard beta-diversity analyses (nonmetric multidimensional scaling/Principal Coordinates Analysis nMDS/PCoA), while raw counts were used for RC_bray null models. Community dissimilarity between samples was calculated using the Bray–Curtis distance metric. To visualize the reproducibility among biological triplicates, hierarchical clustering was performed on the Bray–Curtis dissimilarity matrix using the unweighted pair group method with arithmetic mean (UPGMA), and the results were displayed as a clustergram (Fig. 2).

To assess the homogeneity of group variances, PERMDISP (betadisper) [27] analysis was performed on the stable phase communities. Procrustes analysis [28] was used to statistically test the congruence between the ordinations of main and rare taxa.

**Figure 3:**
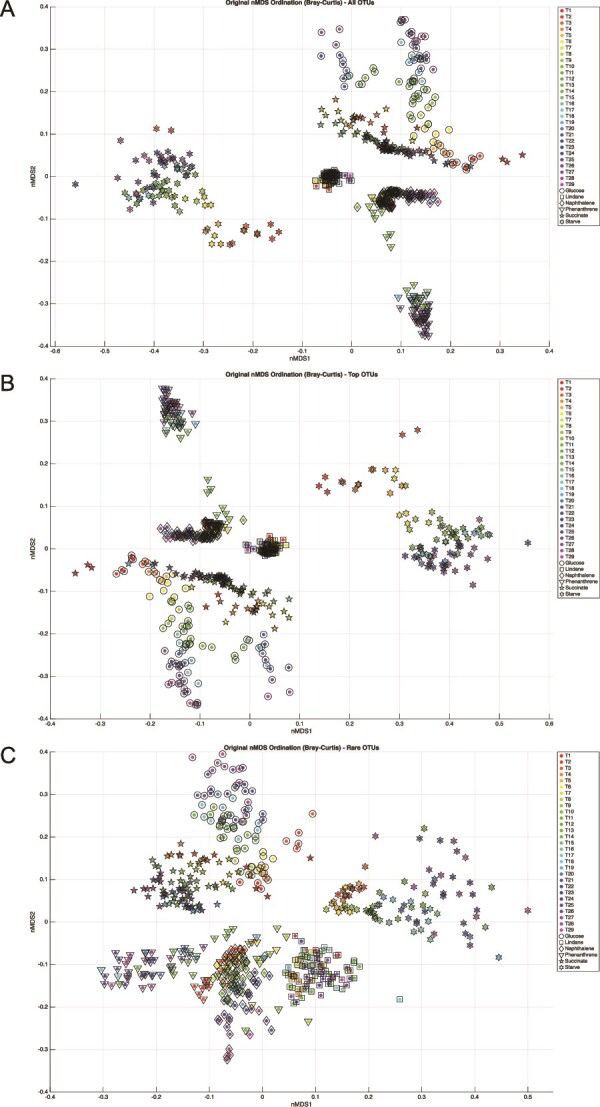
Decoupled successional trajectories of dominant and rare subcommunities. Nonmetric multidimensional scaling (nMDS) plots illustrate the temporal evolution of community structure based on Bray–Curtis dissimilarity. Each point represents a sample at a specific time point. (A) Overall community: Shows the trajectory for all bacteria. (B) Main taxa (>1% abundance): The community structure diverges significantly only under the influence of glucose and phenanthrene. (C) Rare taxa (<0.1% abundance): The rare biosphere exhibits unique and divergent successional trajectories for all carbon source conditions, indicating that it is governed by a distinct set of ecological pressures.

To investigate the dynamics of different community fractions, OTUs were partitioned into “main” (>1% mean relative abundance across all samples) and “rare” (<0.1% mean relative abundance) subcommunities. nMDS was then used to visualize the successional trajectories of the entire community, as well as the “main” and “rare” subcommunities, based on their respective Bray–Curtis dissimilarity matrices (Fig. 3).

To infer interspecies interaction networks while accounting for temporal autocorrelation, we employed Extended Local Similarity Analysis (eLSA) [29] instead of simple correlation. The algorithm was executed with 1000 permutations and a maximum time delay of 3 time points. Additionally, we classified interactions as “global” (significant across the entire time series) or “local only” (significant only during specific temporal windows).

Finally, to identify taxa statistically associated with specific carbon sources independent of temporal autocorrelation, we performed Indicator Species Analysis (IndVal) [30, 31]. Taxa were considered significant indicators if they possessed an IndVal statistic >0.5 and a *P*-value < .05.

## Results and discussion

### Carbon source consumption and bacterial growth

To assess the impact of substrate bioavailability, we tracked the degradation of various carbon sources and the corresponding bacterial population dynamics over a 14-day incubation. The rate of carbon source consumption varied dramatically among the treatments (Fig. 1). Readily metabolizable substrates (glucose, succinate) supported rapid growth and high density, whereas aromatic hydrocarbons showed a ~6-day lag phase (Fig. 1B, Supplementary Fig. S2). This suggests that their degradation requires a more complex metabolic apparatus or an initial adaptation period for the community. The organochlorine pesticide γ-HCH proved highly recalcitrant, with its concentration remaining near initial levels throughout the 14-day experiment. Analysis of TOC in the supernatant further confirmed these trends (Supplementary Fig. S3). Notably, while the insoluble phenanthrene itself was not measured, the analysis revealed a rise in soluble TOC in these cultures, corresponding to the production of metabolic intermediates like 1-naphthol (Fig. S4) and providing direct evidence of biotransformation.

Bacterial population density, quantified via 16S rRNA gene copy numbers, was positively associated with substrate bioavailability (Fig. 1 and Fig. S5). Cultures supplemented with glucose maintained the highest and most stable population density (~9.5 log₁₀ copies/ml), while succinate, naphthalene, and phenanthrene supported intermediate densities. The population dynamics in the aromatic hydrocarbon treatments differed: the naphthalene culture declined after a brief growth phase, likely due to substrate limitation or toxic intermediate accumulation, whereas the phenanthrene culture decreased for ~4 days before rebounding to ~8.2 log₁₀ copies/ml. As expected, populations under γ-HCH and starved conditions declined steadily. However, the starved community reached a lower final density (~5.0 log₁₀ copies/ml) than the γ-HCH community (~6.0 log₁₀ copies/ml). This counterintuitive result suggests that γ-HCH toxicity, while detrimental overall, may lyse sensitive cells, providing a steady stream of necromass that supports a slightly larger scavenging population than starvation alone. This highlights the critical role of internal nutrient cycling in communities under severe stress.

Analysis of IC provides a potential explanation for why succinate, a readily metabolized substrate, supported a lower population density than glucose. While IC levels remained stable at ~50 mg/l in most treatments, they increased markedly in the succinate cultures within the first 2 days, stabilizing at ~250 mg/l (Fig. S3). This rapid IC accumulation, likely resulting from intense substrate mineralization, suggests a secondary stress (e.g. potential pH change or carbonate toxicity) that may have limited the community’s final carrying capacity.

### Deterministic selection outweighs stochastic effects

Analysis of biological triplicates revealed minimal Bray–Curtis dissimilarity (<0.2) and consistently high mean community overlap (approx. 0.8–0.9, Fig. S6), indicating that the carbon source exerted a dominant selective pressure (Fig. 2). While glucose and phenanthrene drove the assembly of distinct consortia, succinate, naphthalene, and γ-HCH selected for relatively similar communities.

Quantitative analysis revealed significant differences in multivariate dispersion (PERMDISP P = 3.2e-20). Communities on complex substrates were highly constrained (e.g. phenanthrene dispersion = 0.07), whereas starved (0.23) and glucose (0.21) conditions showed significantly higher stochastic variation. Despite these differences in dispersion, ANOSIM confirmed that the carbon source remained the primary determinant of community structure (R ≈ 1.0; Fig. S6).

The mechanism driving this selection appears to be environmental filtering. We observed significant phylogenetic clustering (SES.MPD < 0) and higher-than-expected niche overlap among dominant OTUs (Fig. S6), implying that substrates select for phylogenetically related organisms sharing specific metabolic traits rather than driving competition-based exclusion.

Quantitative partitioning of ecological processes using the Stegen *et al*. null model confirmed that assembly was dominated by homogeneous selection (73.75% of pairwise comparisons). Stochastic processes played a secondary role, with dispersal limitation contributing 19.35% and ecological drift accounting for only 3.83% (Table 1). Crucially, time-resolved analysis (Fig. S7) linked the deterministic filter strictly to active metabolism: stochastic signals emerged only in the absence of a selective resource (starved control), after substrate depletion (glucose), or during the lag phase (phenanthrene). Thus, metabolic activity overrides stochastic drift to enforce distinct, reproducible community structures.

**Table 1:** Quantitative partitioning of ecological assembly processes (null model analysis).

	Within_Replicates (%)			Between_CS (%)		
Process	All	Main	Rare	All	Main	Rare
Variable selection	1.53	22.03	0.77	20.82	29.76	8.28
Homogeneous selection	73.75	0	86.97	2.04	0	42.58
Dispersal limitation	19.35	19.73	2.3	76.73	58.67	45.72
Homogeneous dispersal	1.53	46.36	0.19	0.03	2.96	0
Ecological drift	3.83	11.88	9.77	0.38	8.61	3.42

The table summarizes the relative contribution (%) of deterministic and stochastic processes governing community turnover, calculated using the Stegen *et al*. null model framework. Comparisons were performed Within Replicates (to assess reproducibility) and between carbon sources (to assess treatment effects). Within replicates: High values for homogeneous selection (73.75% overall) confirm that community assembly was highly reproducible. However, assembly mechanisms were fundamentally decoupled between fractions: main taxa (>1%) were driven primarily by homogenizing dispersal (46.36%), reflecting the persistence of shared generalist taxa from the inoculum, whereas rare taxa (<0.1%) were overwhelmingly governed by homogeneous selection (86.97%). This reframes the rare biosphere not as a stochastic “long tail” but as a reservoir subject to strict environmental filtering. Between carbon sources (Between_CS): The ecological drivers shift toward dispersal limitation (76.73%) and variable selection (20.82%). This confirms that the observed beta-diversity between treatments is driven by the specific selective pressure of each substrate (variable selection) combined with the lack of exchange between closed microcosms (dispersal limitation).

### Carbon source drives community composition and succession

#### Overall community structure

The carbon source profoundly dictated the final composition of the bacterial consortia (Fig. 1C). While a core set of genera, including *Bacillus* and *Paenarthrobacter*, was present in all samples, their abundance varied. The relative abundance of other key taxa was strongly substrate-dependent; below are some of the observed taxa (association validated by Indicator Species Analysis, IndVal in parentheses, *P*-value < .05):

- Glucose: Enriched for *Mucilaginibacter gossypiicola* (0.95) and *Chryseobacterium gleum* (0.72).
- Succinate: Enriched for *Brevundimonas halotolerans* (0.88) and *Delftia acidovorans* (0.52).
- Naphthalene: Dominated by a *Segetibacter* species (0.99) and a *Flavisolibacter* species (0.51).
- Phenanthrene: Characterized by a massive enrichment of *Sphingobium cupriresistens* (0.83) (21.6% of total abundance, see Table 2), a known degrader of aromatic compounds, and *Cupriavidus metallidurans* (0.88).
- γ-HCH: Showed high abundance of *Flavobacterium lindanitolerans* (10.0%) [IndVal 0.42, not a statistically significant indicator by the strict definition, but still abundant (10%)] a species named for its ability to tolerate or degrade γ-HCH [32]. Crucially, this OTU was also abundant in the starved condition (4.54%), suggesting its ecological strategy is one of broad stress tolerance and likely scavenging, rather than specific γ-HCH metabolism. This enrichment is particularly intriguing given that significant degradation of γ-HCH was not observed in our experiment. While this may suggest the genetic potential for degradation exists but was not fully expressed under these specific conditions, other explanations are also plausible. For instance, *F. lindanitolerans* may not be consuming γ-HCH directly but rather thriving as a scavenger by efficiently feeding on the necromass of other bacteria that are less tolerant of the toxic conditions. Alternatively, there may have been trace-level degradation below the analytical detection limit, providing just enough resource to support this specialized population. In either case, this demonstrates a powerful deterministic selection for organisms best adapted to survive under severe stress.

**Table 2:** Representative ecological generalists and specialists demonstrating niche partitioning and functional guilds.

Ecological strategy	OTU ID	Class	Species	rrnDB 16S copies (bold exact match rrnDB)	Total abundance (log10)	Glucose	Succinate	Phenanthrene	Naphthalene	γ-HCH	Starve
**Ecological generalists**											
	OTU_16	Bacilli	*Neobacillus drentensis*	**20**	9.7	26.39%	19.31%	15.39%	**27.44%**	16.63%	7.84%
	OTU_32	Actinomycetes	*Arthrobacter ramosus*	6	9.2	**12.58%**	5.50%	3.45%	7.66%	6.93%	0.12%
	OTU_31	Alphaproteobacteria	*Sphingomonas kyeonggiensis*	2	8.9	1.21%	**3.53%**	2.01%	3.51%	2.72%	0.95%
	OTU_17	Betaproteobacteria	*Acidovorax delafieldii*	**4**	8.8	4.56%	1.36%	2.44%	4.27%	**5.98%**	5.59%
**Ecological specialists**											
Glucose specialists	OTU_12	Sphingobacteriia	*Mucilaginibacter gossypiicola*	3	9.0	**7.38%**	0.00%	0.10%	0.09%	0.04%	0.20%
	OTU_50	Flavobacteria	*Chryseobacterium gleum*	**6**	8.9	**5.26%**	0.39%	0.44%	0.52%	0.07%	0.07%
Succinate specialists	OTU_70	Alphaproteobacteria	*Sphingopyxis panaciterrae*	1	7.8	0.07%	**2.20%**	0.09%	0.16%	0.08%	0.19%
	OTU_28	Alphaproteobacteria	*Brevundimonas halotolerans*	2	7.5	0.00%	**1.42%**	0.03%	0.06%	0.00%	0.00%
Phenanthrene specialists	OTU_3	Alphaproteobacteria	*Sphingobium cupriresistens*	2	8.7	0.00%	0.00%	**21.59%**	0.00%	0.00%	0.01%
	OTU_77	Betaproteobacteria	*Cupriavidus metallidurans*	**4**	8.0	0.03%	0.03%	**3.88%**	0.01%	0.01%	0.00%
Naphthalene specialists	OTU_129	Flavobacteria	*Chryseobacterium oncorhynchi*	5	7.6	0.01%	0.00%	0.33%	**0.64%**	0.01%	0.08%
	OTU_23	Sphingobacteriia	*Flavisolibacter sp. (GQ339898_s)*	3	7.9	0.00%	0.00%	0.82%	**1.20%**	0.06%	0.30%
γ-HCH specialists	OTU_42	Flavobacteria	*Flavobacterium lindanitolerans*	5	8.6	0.67%	3.76%	1.07%	0.69%	**9.95%**	4.54%
	OTU_63	Gammaproteobacteria	*Lysobacter soli*	**2**	7.4	0.04%	0.19%	0.11%	0.19%	**0.79%**	0.11%
Starvation specialists	OTU_13	Oligoflexia	*Bdellovibrio sp. (DQ833495_s)*	2	8.2	0.00%	0.00%	0.03%	0.00%	0.01%	**5.42%**
	OTU_18	Flavobacteria	*Flavobacterium anhuiense*	**5**	8.2	0.39%	0.05%	0.04%	0.06%	0.29%	**3.00%**

The table displays the absolute abundance (as a percentage of the total bacterial community) for key OTUs representative of different ecological strategies. Ecological generalists form a resilient “core” community, maintaining a notable presence across multiple environments. Ecological specialists are highly enriched in specific niches, often forming functional guilds of different taxa that respond to the same environmental filter. The log10 abundance column indicates the log10-transformed total 16S rRNA sequence count for each OTU across all samples, representing its overall amplicon footprint in the environment. Note that due to intrinsic 16S rRNA gene copy number variations between taxa, this value represents sequence dominance rather than absolute cellular biomass. The top value for each OTU is highlighted as bolded boxes. The full lists of the top 100 generalists and specialists are available in Tables S2 and S3.

#### Bacterial succession dynamics

A community-level view of succession, assessed through diversity and richness indices, revealed distinct responses to the different carbon sources (Fig. S8, Fig. S9). The initial soil extract inoculum maintained high diversity and richness during pre-incubation (negative time points). Following the addition of substrates at Day 0, most treatments experienced a sharp drop in diversity and richness, a classic bottleneck effect caused by strong selection for a few fast-growing specialists. However, a key exception was the γ-HCH culture; under severe toxic stress rather than resource abundance, it maintained its initial diversity and richness without a significant drop, suggesting a different selective pressure that did not favor a single dominant “winner.”

In the later stages of the experiment (after Day 8), the communities stabilized at distinct diversity (Simpson’s Dr) and richness (Margalef’s) levels. The diversity ranking was clearly stratified: glucose cultures recovered to sustain the highest diversity, followed by γ-HCH, then naphthalene and succinate (which were similar), and finally phenanthrene, which exhibited the lowest diversity due to its domination by a single specialist. Richness, however, told a different story. While most cultures converged to a similar level of richness, the succinate culture consistently maintained the lowest number of taxa, suggesting that the secondary stress induced in that environment may have driven some rarer species to local extinction.

We also estimated 16S rRNA operon copy numbers (Table 2) using the rrndb database [33]. Early successional drivers like *Neobacillus drentensis* possess high copy numbers (~20 copies), consistent with an r-strategy, whereas stress-tolerant specialists like *Sphingobium* possess low copy numbers (1–2 copies), consistent with K-strategy efficiency. Because taxa with high *rrn* copy numbers generate disproportionately more amplicons per cell, it is important to note that the total 16S sequence abundances observed here represent an ecological “amplicon footprint” rather than absolute cellular biomass. Therefore, life-history strategies (r- vs. K-selection) were inferred strictly from the intrinsic genomic trait (*rrn* copy number) rather than their amplified sequence abundance.

The temporal succession of key bacterial populations revealed classic ecological dynamics (see Fig. S10).

- In glucose cultures, an initial bloom of *N. drentensis* (likely a fast-growing r-strategist from the soil extract) was followed by its decline as glucose was depleted. Subsequently, other genera like *Pseudomonas* and *Chryseobacterium* increased, likely acting as secondary consumers feeding on metabolic byproducts or dead biomass.
- In phenanthrene cultures, a dramatic succession event occurred around Day 5, coinciding with the onset of degradation. The initial dominant, *N. drentensis*, was rapidly replaced by *S. cupriresistens* and *Pseudomonas*, specialists in aromatic hydrocarbon degradation.
- In succinate cultures, *N. drentensis* remained dominant, but its proportion fluctuated, indicating more complex co-existence and competitive dynamics with other genera like *Arthrobacter*.
- In starved and γ-HCH cultures, the initial dominant taxa declined, while more resilient bacteria (e.g. *Acidovorax*) persisted at low levels, showcasing survival strategies (K-strategists) under nutrient-poor conditions.

#### Structure of interspecies interaction networks

eLSA revealed that the underlying structure of bacterial interactions was profoundly shaped by the carbon source, differing not just in connectivity but in temporal stability (Table 3, Tables S4 and S5, Fig. S11).

**Table 3:** Interspecies interaction network topology (eLSA).

Correlations	Positive	Negative	$\frac{\mathrm{Positive}}{\mathrm{Negative}}$
Glucose	**37 230**	**25 281**	1.47
γ-HCH	6511	4326	1.51
Naphthalene	21 516	11 540	1.86
Phenanthrene	20 266	6761	**3.00**
Succinate	19 507	9565	2.04
Starve	19 477	13 576	1.43

Summary of significant pairwise associations identified using Extended Local Similarity Analysis (*P* < .05, max delay = 3 time points). The positive/negative ratio indicates the balance between potential cooperative (e.g. cross-feeding, syntrophy) and competitive interactions. Bold values indicate the highest count or ratio within each respective column. The phenanthrene treatment exhibits the highest prevalence of positive associations (ratio: 3.00), suggesting that the degradation of complex aromatic substrates relies heavily on cooperative metabolic guilds. In contrast, glucose and starvation treatments display lower ratios (~1.4–1.5), reflecting a tighter balance between interaction and competitive exclusion.

In the glucose treatment, the network was characterized by a high frequency of “global” negative interactions (70% of negative edges). This reflects permanent competitive exclusion, where dominant fast-growers suppress slower taxa throughout the entire incubation in a resource-rich environment. In contrast, positive interactions in complex substrates like Phenanthrene were frequently “local only” (53% of positive edges). This topology suggests transient cooperative relays or metabolic cross-feeding chains where functional guilds replace one another as intermediates are degraded, rather than static mutualisms.

The network topology under stress conditions (Starvation and γ-HCH) provided strong support for the Stress Gradient Hypothesis (SGH) [31, 34]. This theory predicts that in harsh environments, facilitative interactions become more prevalent than competition. While global network statistics (Table 3) indicated that Starvation and γ-HCH maintained high overall competitive pressure comparable to Glucose, time-resolved analysis (Table S5, Fig. S11) uncovered a hidden layer of facilitation predicted by the Stress Gradient Hypothesis (SGH). Specifically, while glucose was dominated by “global” negative interactions (permanent competitive exclusion), Starvation and γ-HCH exhibited a surge in “local” (transient) positive associations (starvation local Pos/Neg ratio ~ 3.0 vs. glucose ~2.4). Figure S11 confirms the rapid onset of these facilitative interactions in stress conditions, suggesting that survival relies on fleeting cooperative events—such as necromass scavenging or detoxification relays [35]—rather than direct resource competition.

### Decoupled dynamics of main and rare bacteria

We separated the community into “main” bacteria (>1% relative abundance) and “rare” bacteria (<0.1% abundance), revealing a striking divergence in their behavior via nMDS analysis (Fig. 3). The successional trajectory of main bacteria mirrored the overall community, with significant divergence observed only in glucose and phenanthrene treatments. Conversely, the rare bacteria displayed unique, divergent trajectories across all conditions, a resolution made possible only by our high-frequency sampling. While Procrustes analysis indicated a significant baseline association between the subcommunities (*P* = .001), the moderate goodness-of-fit (m^2^ = 0.4520) and Euclidean Trajectory Distance analysis (Fig. S12) confirmed distinct pacing: Main taxa stabilized quickly at a distance of ~0.52, whereas rare taxa continued to diverge (>0.66).

This decoupling suggests that the subcommunities are governed by different ecological rules. Main bacteria are strongly selected by primary environmental filters such as temperature [20, 36], pH [37–40], and carbon source (this work, [41–45]). Global Null Model analysis (Table 1) confirms this mechanistic explanation: main taxa were driven by a hybrid assembly process dominated by dispersal limitation (58.67%)—reflecting priority effects in closed systems—with a significant secondary role for variable selection (29.76%). This confirms that while generalists initially adapt to the basal medium, chemically distinct substrates act as precise metabolic filters, forcing community divergence when specialists (e.g. *Sphingobium* in phenanthrene) become dominant.

To distinguish substrate-driven selection from baseline “bottle effects,” we performed a time-resolved null model analysis comparing treatments to the starved control (Fig. S13). For main bacteria, substrate addition acted as an immediate primary filter. In glucose and γ-HCH treatments, variable selection peaked early, driven by rapid resource uptake or toxicity. In phenanthrene, selection rose to ~60% around Day 6, aligning strictly with the onset of degradation. In contrast, succinate treatments showed lower variable selection. As a central tricarboxylic acid (TCA) cycle intermediate, succinate utilization traits are phylogenetically widespread, imposing less restrictive pressure than the strong r-selection of glucose (which often triggers carbon catabolite repression [46]) or the toxicity of γ-HCH. This explains why trajectories for succinate, lindane, and naphthalene remained clustered near the origin in the nMDS (Fig. 3B), while glucose and phenanthrene drove communities into distinct ordination spaces.

The rare biosphere appears to respond to a different suite of drivers. Globally, its assembly was dominated by dispersal limitation (45.72%) and homogeneous selection (42.58%), suggesting it functions as a “seed bank” of stress-tolerant organisms. Instead of being shaped purely by stochasticity, the rare subcommunity likely responds to environmental filters less critical for dominant taxa, such as trace minerals, secondary metabolites, or heightened susceptibility to predation pressure and viral lysis.

Our time-resolved analysis (Fig. S13) resolves the apparent contradiction between the global stochasticity of the rare biosphere and its continuous visual divergence in the nMDS. During early incubation, the rare subcommunity is indeed governed by homogeneous selection and dispersal limitation. Yet, in late successional stages—particularly with complex substrates like phenanthrene—variable selection surges to ~50%. This shift suggests the rare biosphere is a “latent responder,” reacting not to the initial substrate, but to downstream successional triggers such as toxic intermediates or necromass. This refines the dichotomy proposed by Jiao *et al*. [35, 47] who suggested rare bacteria are influenced primarily by stochasticity; our data reveal that this “stochasticity” is actually a latent phase that shifts toward deterministic selection once successional triggers are met. This trajectory decoupling is further supported by [48], who demonstrated that specific nutrient regimes can drive the assembly of abundant and rare subcommunities in fundamentally different directions. Under the right pressure, rare members can surge to dominance, as seen with *Sphingobium* here or *Pseudomonas* in our previous work [35].

In summary, this work demonstrates that carbon sources act as powerful deterministic filters shaping successional patterns from primary consumers to specialists. By illustrating these patterns and showing how interaction networks shift from competitive to facilitative along stress gradients, this study reinforces fundamental ecological principles. The most significant finding is the decoupled dynamics of the community: main bacteria are primary consumers shaped by immediate resource availability, while the rare biosphere acts as a dynamic, buffered reservoir that reshapes deterministically in response to downstream triggers. This dataset (SRA accession: PRJNA1299551) offers a valuable resource for future modeling of these core assembly principles.

## Supplementary Material

ycag108_Supplemental_Files

## Data Availability

The 16S rRNA gene amplicon sequencing datasets generated and analyzed during the current study are available in the NCBI Sequence Read Archive (SRA) repository under accession number PRJNA1299551.
