# Supplementary material for: Carbon source acts as a deterministic filter shaping microbial succession and rare-abundant decoupling in soil bacterial communities
Source: ISME Commun. 2026 Apr 20;6(1):ycag108. doi: 10.1093/ismeco/ycag108 (PMC13184967; doi:10.1093/ismeco/ycag108)
Supplement: ycag108_Supplemental_Files [file ycag108_supplemental_files.zip › Stari_Sup_260410_ycag108.pdf]

Supplementary information

Carbon Source Acts as a Deterministic Filter Shaping Microbial Succession and Rare-Abundant Decoupling in Soil Bacterial Communities

Authors: Leonardo Stari[1], Hiromi Kato[1],Kouhei Kishida[1], Yoshiyuki Ohtsubo[1], Michio Kondoh[1] and Yuji Nagata[1]*
Affiliation: [1]Graduate School of Life Sciences, Tohoku University, Katahira 2-1-1, Aoba-ku, Sendai 980-8577, Japan
*Corresponding author's email: aynaga@ige.tohoku.ac.jp

This PDF file includes:

Table S1: Temporal structure of interspecies interactions (eLSA)

Table S2: Temporal stability of interactions (Global vs. Local eLSA)

Table S3&S4: Complete Lists of Top 100 Ecological Generalists (Table S3) and Specialists (Table S4)

Table S5: Quantitative comparison of ecological trajectories and subcommunity partitioning using 97% OTU versus zero-radius Operational Taxonomic Unit (zOTU) bioinformatic pipelines

FIG S1: Differential consumption of carbon sources over time

FIG S2: Species-Level Composition of Bacterial Consortia

FIG S3: Carbon source dictates bacterial population density dynamics

FIG S4: Metabolite tracking confirms phenanthrene-fueled bacterial growth dynamics

FIG S5: Temporal changes in the relative abundance of the most dynamic bacterial genera across the six experimental conditions

FIG S6: Community diversity and richness dynamics

FIG S7: Analysis of Soluble Carbon Pools Reveals Substrate Mineralization and Byproduct Formation

FIG S8: Comprehensive Analysis of Microbial Community Dynamics and Assembly Mechanisms

FIG S9: Pooled rarefaction curves of bacterial communities under different carbon source conditions

FIG S10: Decoupled temporal divergence of Main and Rare bacterial subcommunities

FIG S11: Normalized temporal density of interactions (eLSA)

FIG S12: Temporal dynamics of ecological assembly processes within replicates

FIG S13: Decoupled temporal dynamics of ecological assembly processes for Main vs. Rare subcommunities

Table S1: Temporal structure of interspecies interactions (eLSA). Detailed breakdown of significant pairwise associations ($P < 0.05$) categorized by time lag. Shifts range from -3 to +3 time points (approx. 12 h intervals). Synchronous associations (lag = 0) suggest immediate responses (e.g., shared niche or direct competition), while time-lagged associations reflect delayed dynamics characteristic of cross-feeding or succession. Notably, the Glucose condition exhibits a high frequency of synchronous negative interactions, consistent with rapid competitive exclusion.

eLSA	Positive						
	-3	-2	-1	0	1	2	3
Glucose	1,008	1,834	5,285	20,710	4973	2082	1338
γ-HCH	298	580	768	3,576	719	417	153
Naphthalene	669	1,495	2,848	13,332	1911	792	469
Phenanthrene	561	1,263	2,018	11,992	2072	1581	779
Succinate	651	1,256	2,871	9,725	2773	1411	820
Starve	899	2,384	1,752	10,948	1558	1314	622
eLSA	Negative						
	-3	-2	-1	0	1	2	3
Glucose	483	1,210	3,554	15,750	2754	942	588
γ-HCH	171	308	499	2,509	463	252	124
Naphthalene	230	734	1,302	8,229	721	238	86
Phenanthrene	239	542	835	3,531	855	523	236
Succinate	252	657	1,765	4,643	1433	595	220
Starve	454	1,609	1,368	8,083	1027	582	453

Table S2: Temporal stability of interactions (Global vs. Local eLSA). Classification of significant interactions based on their temporal persistence. "Global" interactions are significant across the entire time series (indicating permanent relationships, such as competitive exclusion), while "Local" interactions are significant only during specific temporal windows (indicating transient relationships, such as successional metabolic relays). Glucose: Characterized by a high density of Global Negative interactions, consistent with permanent suppression of slower growers by dominant generalists. Phenanthrene: Exhibits a high count of Local Positive interactions, supporting the hypothesis of transient cooperative relays required for the stepwise degradation of complex hydrocarbons.

eLSA interactions	**Local only**		**Global**	
	Positive	Negative	Positive	Negative
Glucose	42,753	17,499	54,818	40,560
Lindane	24,227	10,271	11,881	7,536
Naphthalene	32,967	12,391	35,484	20,524
Phenanthrene	42,191	9,611	36,862	12,737
Succinate	26,715	9,480	27,634	15,890
Starve	51,416	16,985	33,158	22,203

Supplementary Tables S3 & S4: Complete Lists of Top 100 Ecological Generalists and Specialists.

These tables provide the full data for the top 100 most abundant OTUs classified as ecological generalists (Table S3) and specialists (Table S4). Available as supplementary excel file generalist_specialists.xlsx

Classification Method: Taxa were classified based on their distribution across the six carbon source conditions (Glucose, Succinate, Phenanthrene, Naphthalene, γ-HCH, and Starve). Classification was performed using the Normalized Relative Abundance metric.

- **Generalists (Table S3):** Defined as OTUs exhibiting high normalized relative abundance (value > 0.5) in **three or more** conditions.
- **Specialists (Table S4):** Defined as OTUs exhibiting high normalized relative abundance (value > 0.5) in only **one or two** conditions.

Column Definitions:

- **OTU ID, Class, Species:** Taxonomic identification of the Operational Taxonomic Unit.
- **log10 total abundance:** The log10-transformed total 16S rRNA gene copy number for the OTU summed across all samples, indicating its overall biomass.
- **Normalized Relative Abundance (columns 5-10, 0-1 scale):** The abundance of an OTU within a condition, scaled from 0 to 1, where 1.0 represents the maximum abundance achieved by that specific OTU across any of the six conditions. This metric was used for the classification of taxa into generalist/specialist categories.
- **Absolute Abundance (% columns 11-16):** The OTU's total copy number within a given condition divided by the total bacterial copy number for that entire condition. This metric represents the OTU's proportional contribution to the overall community structure and is the value displayed in the main text's Table 1.

Table S5: Quantitative comparison of ecological trajectories and subcommunity partitioning using 97% OTU versus zero-radius Operational Taxonomic Unit (zOTU) bioinformatic pipelines. To confirm the robustness of our ecological conclusions, raw sequencing reads were processed in parallel using a 97% identity clustering pipeline (vsearch) and an zOTU pipeline (unoise3). Mantel tests comparing the resulting Bray-Curtis dissimilarity matrices demonstrate near-perfect correlation ($r = 0.995$, $p < 0.001$) at the whole-community level, confirming that global successional trajectories are independent of the clustering method. Furthermore, the distinct temporal dynamics of the "Main" (>1%) and "Rare" (<0.1%) subcommunities remain strongly correlated between pipelines. However, applying the >1% relative abundance threshold to the zOTU dataset captured only ~8% of the total sequence abundance, compared to ~88% in the OTU dataset. This indicates that the zOTU approach heavily fractures dominant functional populations into minor strain-level variants (likely exacerbated by intragenomic 16S rRNA operon heterogeneity, such as the ~20 copies present in Neobacillus). Because this study aims to track the macroscopic ecological succession of dominant functional groups rather than microdiversity, the 97% OTU framework was retained as the most cohesive operational unit, while this zOTU analysis confirms that the central conclusion—the decoupling of rare and abundant subcommunities—is mathematically robust.

Metric / Parameter	97% OTU Pipeline (`vsearch`)	zOTU Pipeline (`unoise3`)
**Total Features (Global)**	5472	6997
**Total "Main" Features (>1% rel. abundance)**	137	50
**Sequence Abundance Captured by "Main" Features**	~88%	~8%
**Total "Rare" Features (<0.1% rel. abundance)**	4882	4185
**Mantel Test: Whole Community (Bray-Curtis)**	—	r = 0.995, $p < 0.001$
**Mantel Test: "Main" Subcommunity (Bray-Curtis)**	—	r = 0.848, $p < 0.001$
**Mantel Test: "Rare" Subcommunity (Bray-Curtis)**	—	r = 0.857, $p < 0.001$

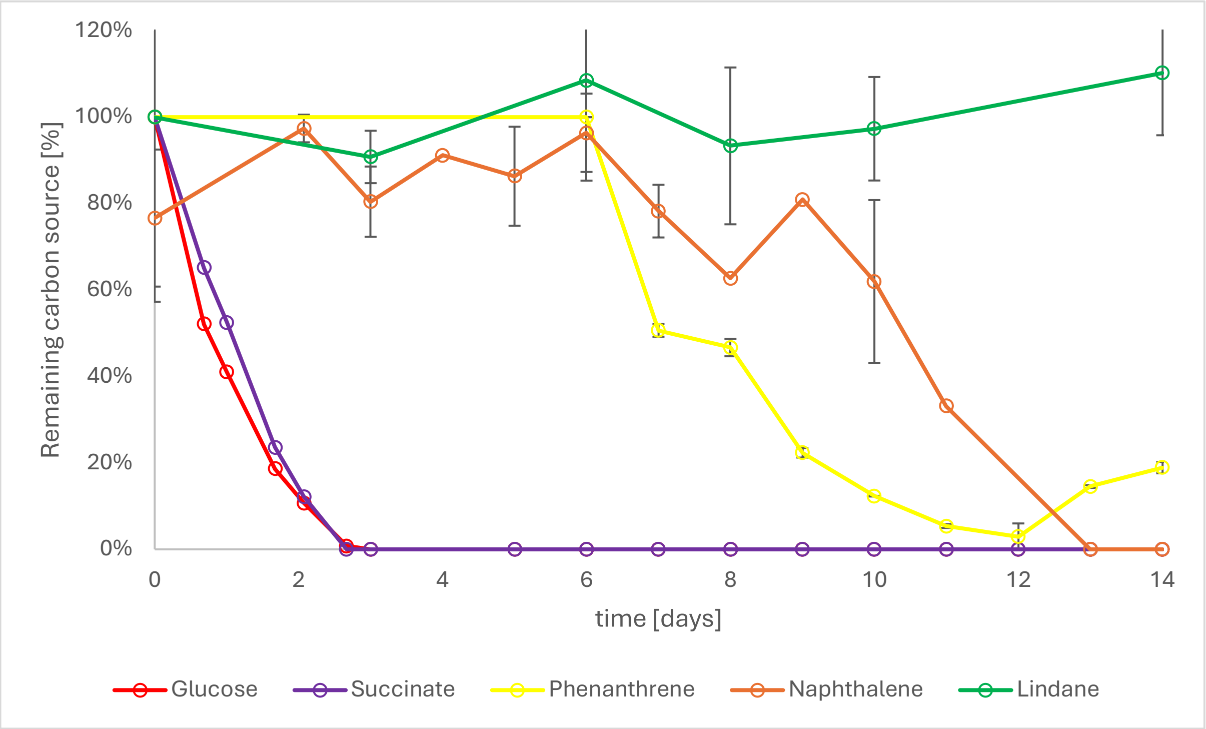

Figure S1: Differential consumption of carbon sources over time. The plot shows the percentage of the initial carbon source concentration remaining in the culture medium over the 14-day experiment. Easily metabolizable substrates (glucose, succinate) were rapidly depleted, while polycyclic aromatic hydrocarbons (naphthalene, phenanthrene) were consumed more gradually. The organochlorine γ-HCH remained recalcitrant. Error bars show standard deviation for phenanthrene, naphthalene and γ-HCH- samples (n=3), glucose and succinate n=1.

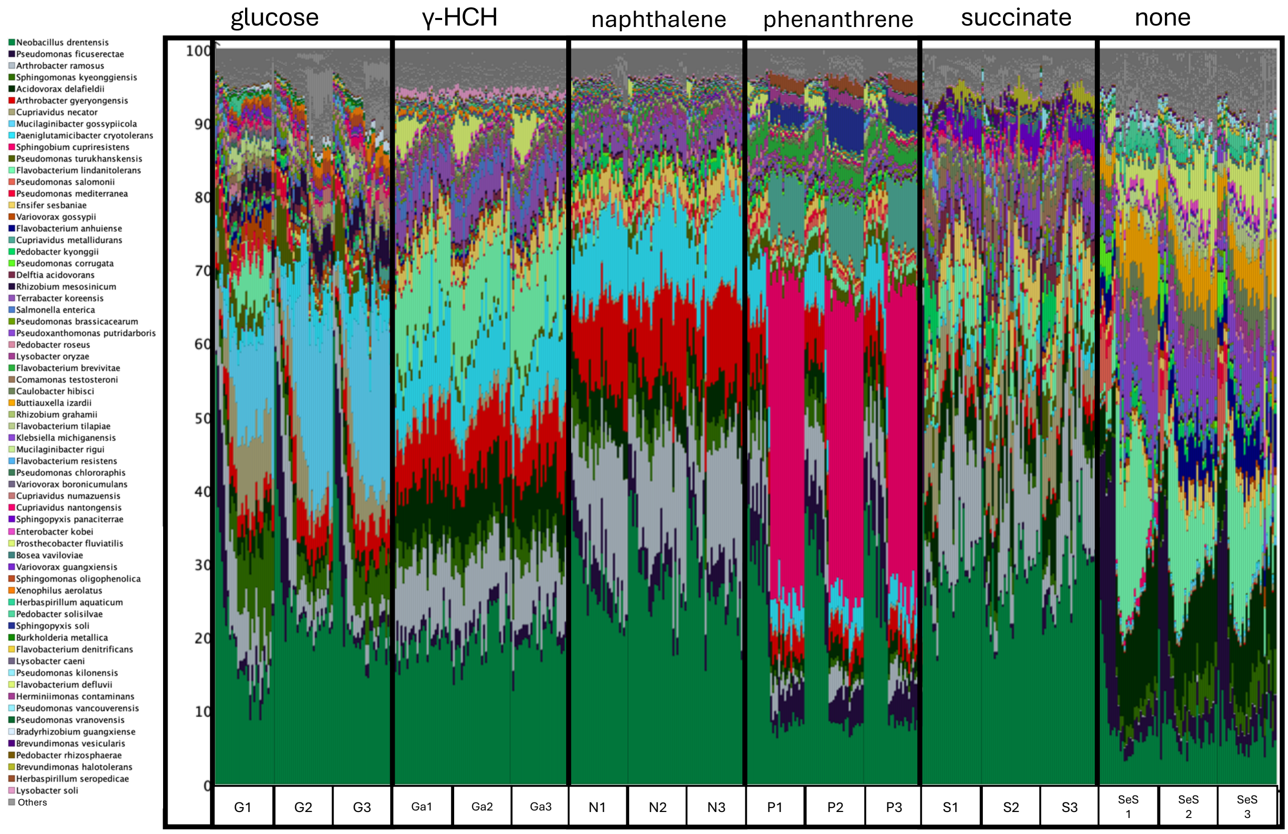

Figure S2: Species-Level Composition of Bacterial Consortia. Stacked bar chart showing the relative abundance of the top 65 OTUs, plus an "Others" category for all remaining taxa, over 14 days (29 samples per biological triplicate). Each panel represents a biological triplicate for a given carbon source. Distinct and reproducible patterns are evident for each substrate.

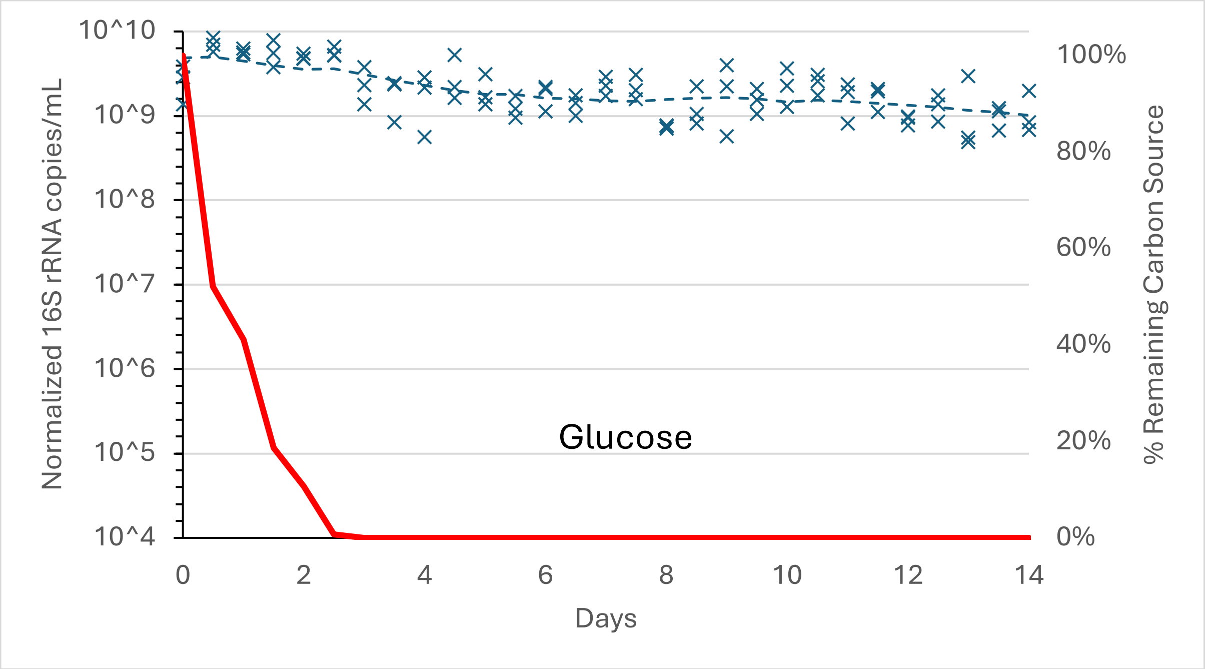
Glucose
Normalized 16S rRNA copies/mL
10^10
10^9
10^8
10^7
10^6
10^5
10^4
% Remaining Carbon Source
100%
80%
60%
40%
20%
0%
0
2
4
6
8
10
12
14
Days

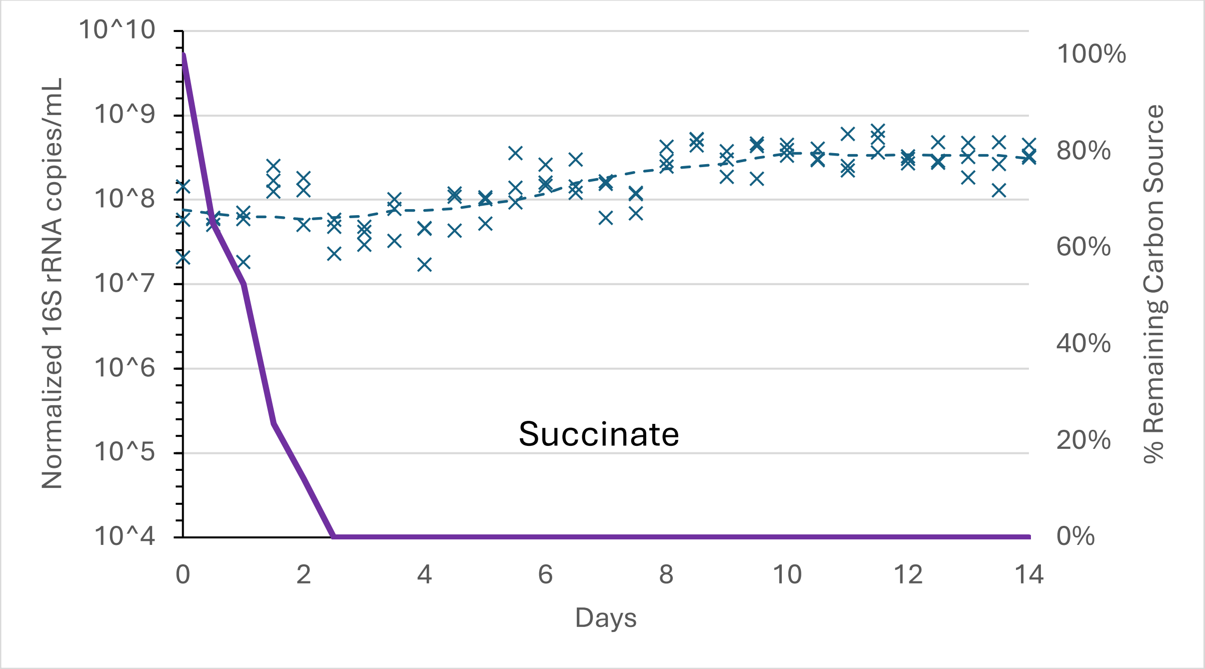
Succinate
Normalized 16S rRNA copies/mL
10^10
10^9
10^8
10^7
10^6
10^5
10^4
% Remaining Carbon Source
100%
80%
60%
40%
20%
0%
0
2
4
6
8
10
12
14
Days

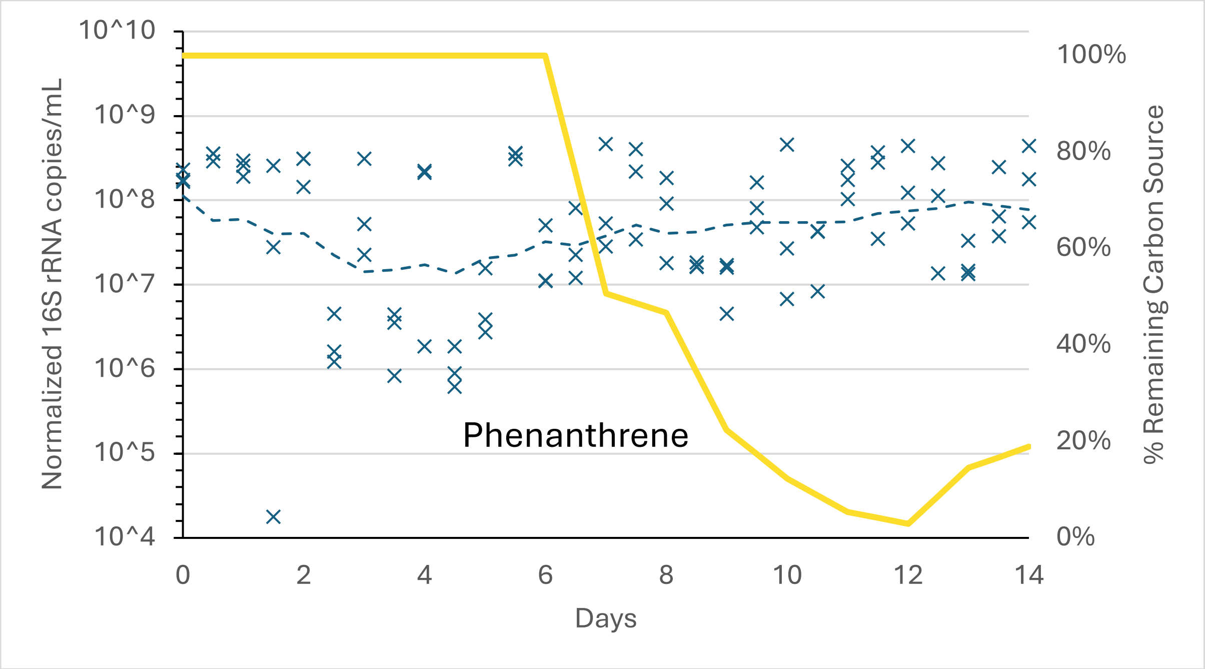
10^10
10^9
10^8
10^7
10^6
10^5
10^4
Normalized 16S rRNA copies/mL
100%
80%
60%
40%
20%
0%
% Remaining Carbon Source
0
2
4
6
8
10
12
14
Days
Phenanthrene

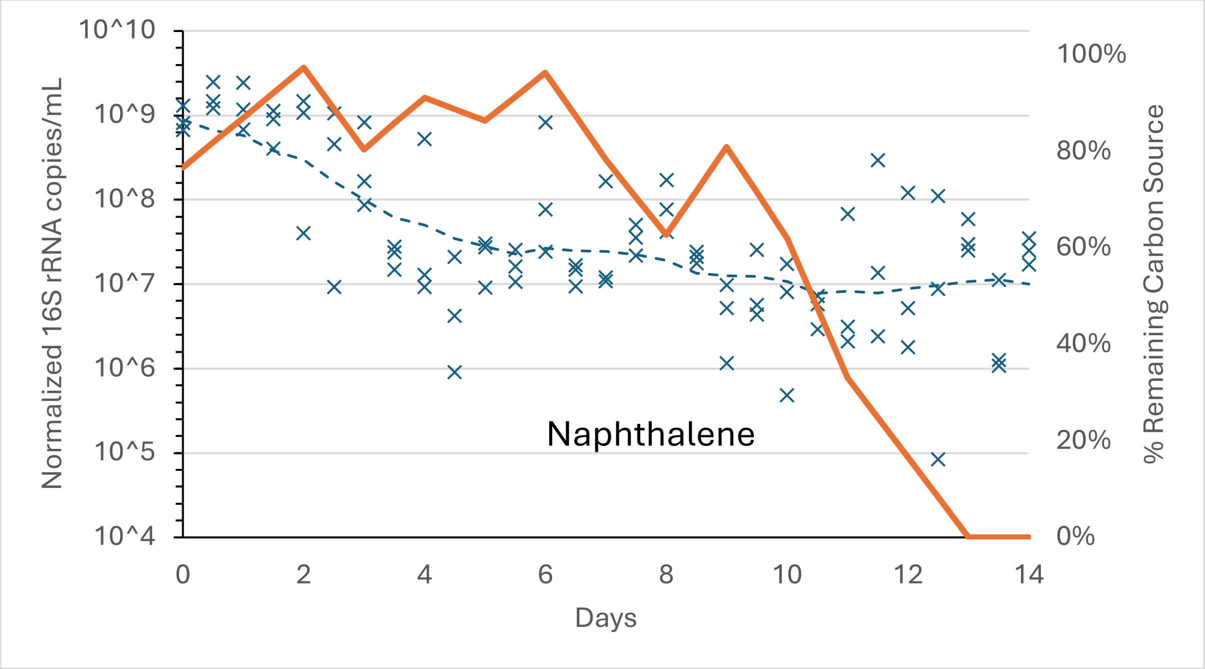
10^10
10^9
10^8
10^7
10^6
10^5
10^4
Normalized 16S rRNA copies/mL
100%
80%
60%
40%
20%
0%
% Remaining Carbon Source
0
2
4
6
8
10
12
14
Days
Naphthalene

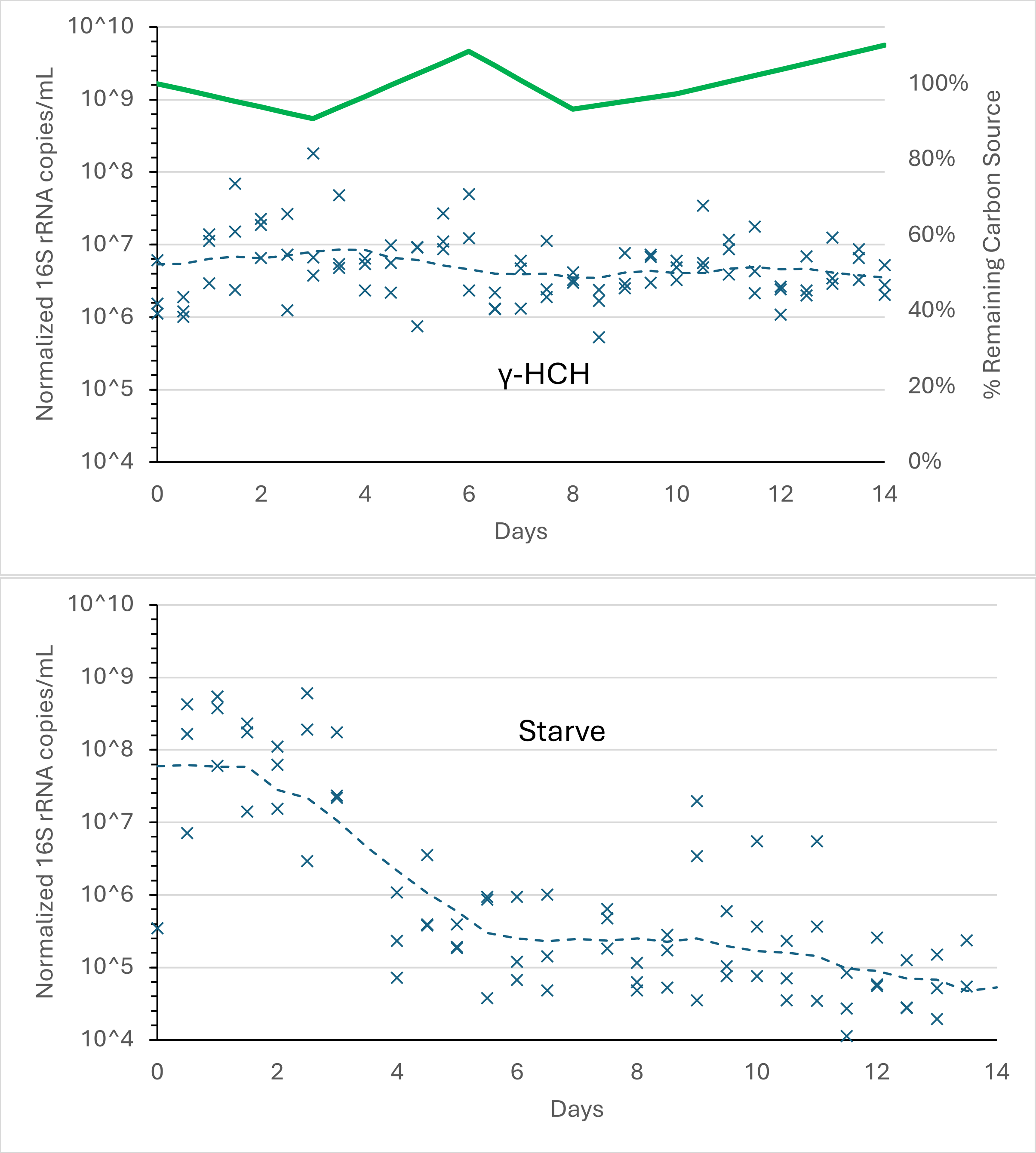

Figure S3: Carbon source dictates bacterial population density dynamics. Temporal changes in total bacterial abundance, measured as log10 16S rRNA gene copies/mL, are shown for each carbon source condition. Each panel displays the change in a specific substrate concentration (solid line, right y-axis) alongside the corresponding bacterial growth (x indicates individual data points, dashed line shows a 5 points rolling window average, left y-axis). Glucose supported the highest and most stable population density, while populations in γ-HCH and starved conditions steadily declined.

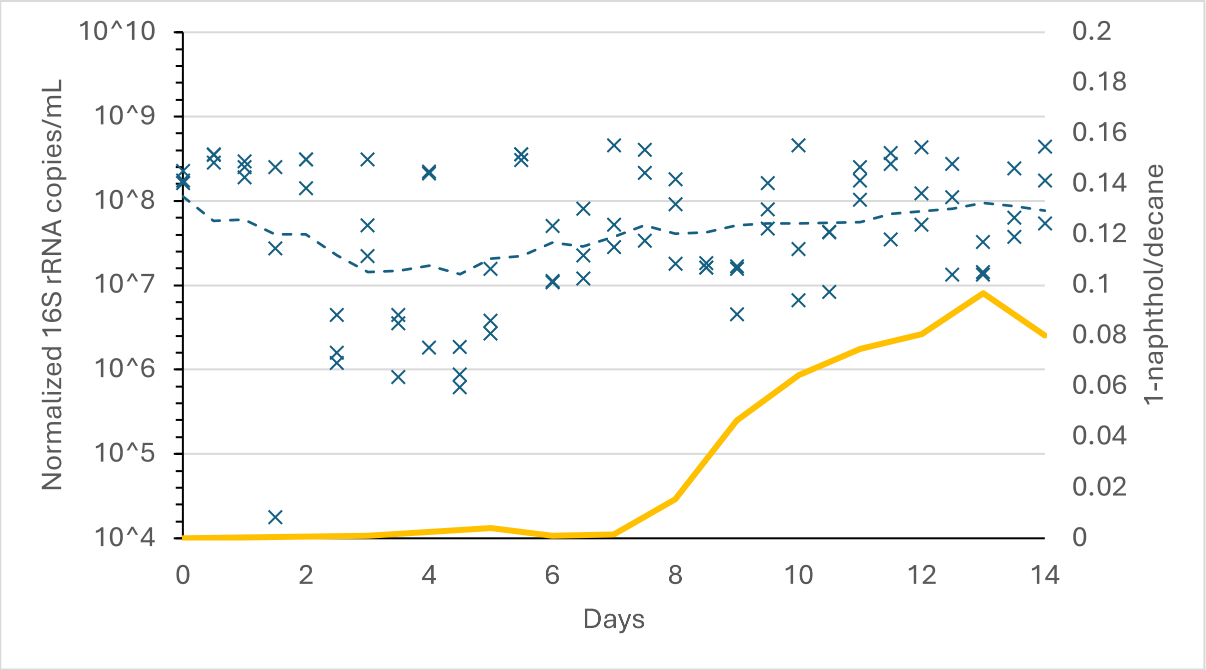

Figure S4: Metabolite tracking confirms phenanthrene-fueled bacterial growth dynamics. Bacterial abundance (x indicates individual data points, dashed line shows a 5 points rolling window average, left y-axis) and the relative concentration of the degradation intermediate 1-naphthol (orange line, right y-axis) were monitored in phenanthrene-amended cultures. Following a community bottleneck and population decline (days 4-8), bacterial growth commenced. This growth was subsequently followed by the accumulation of 1-naphthol, a key intermediate in phenanthrene metabolism. This temporal coupling provides direct biochemical evidence that the observed population increase is fueled by the biotransformation of phenanthrene. Bacterial abundance is measured in 16S rRNA gene copies/mL, and 1-naphthol is represented as a peak area ratio to a decane internal standard.

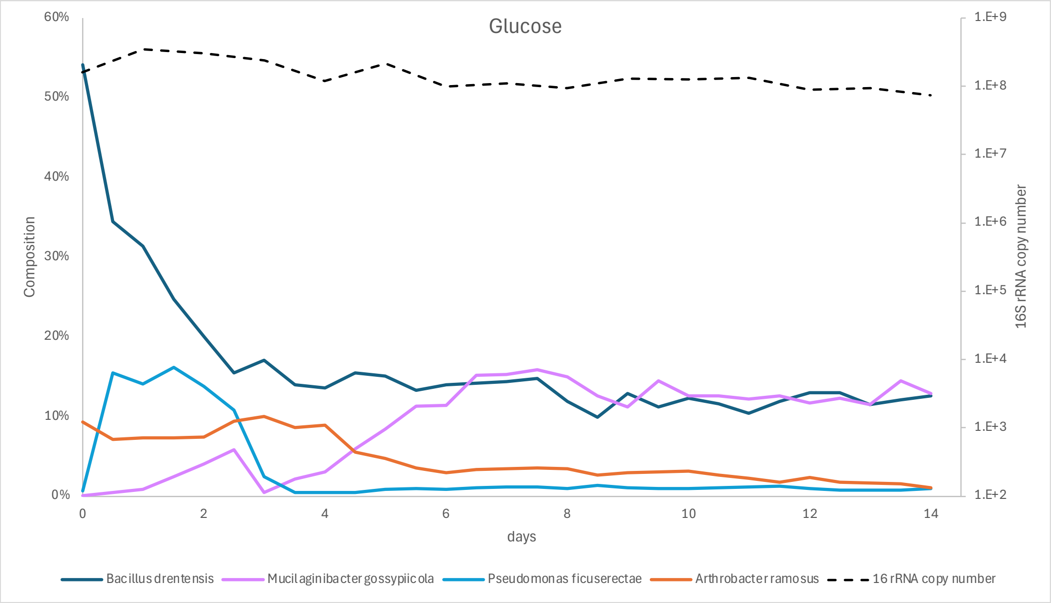
Glucose
Composition
16S rRNA copy number
days
60%
50%
40%
30%
20%
10%
0%
1.E+9
1.E+8
1.E+7
1.E+6
1.E+5
1.E+4
1.E+3
1.E+2
0
2
4
6
8
10
12
14
Bacillus drentensis
Mucilaginibacter gossypiicola
Pseudomonas ficuserectae
Arthrobacter ramosus
16 rRNA copy number

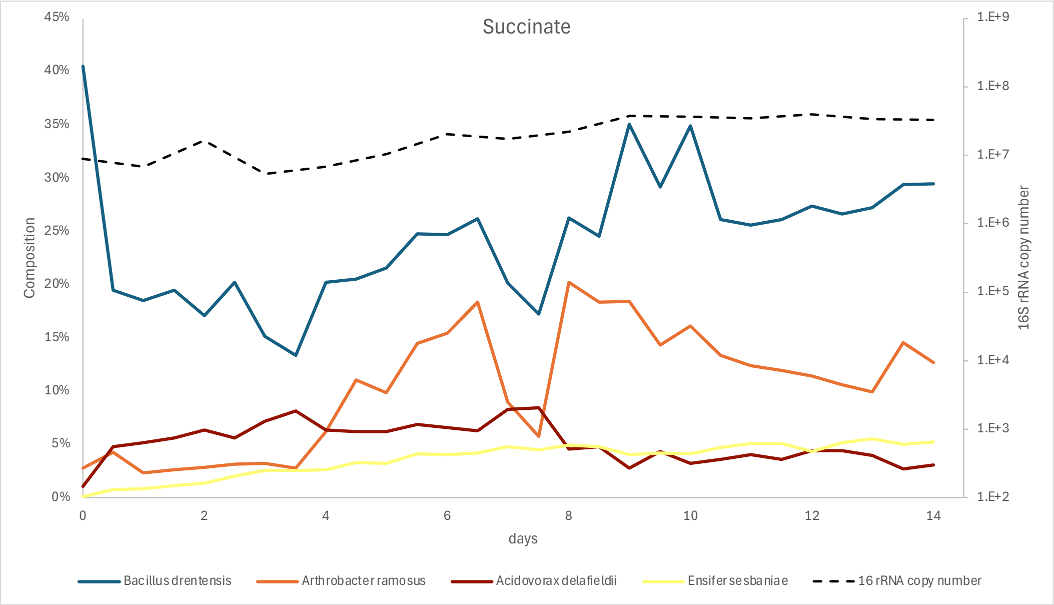
Succinate
Composition
16S rRNA copy number
days
45%
40%
35%
30%
25%
20%
15%
10%
5%
0%
1.E+9
1.E+8
1.E+7
1.E+6
1.E+5
1.E+4
1.E+3
1.E+2
0
2
4
6
8
10
12
14
Bacillus drentensis
Arthrobacter ramosus
Acidovorax delafieldii
Ensifer sesbaniae
16 rRNA copy number

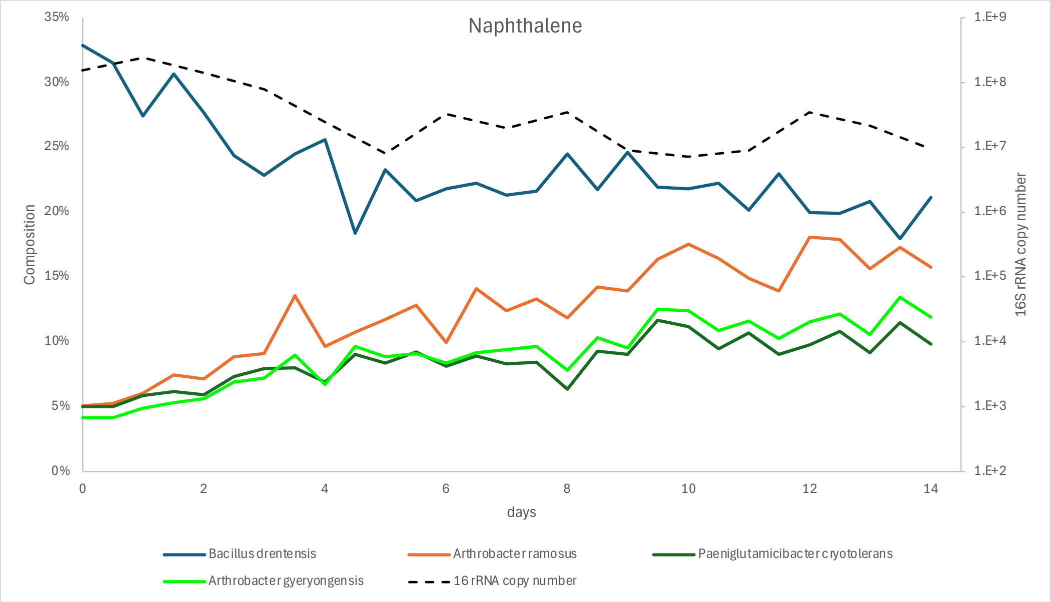
Naphthalene
Composition
16S rRNA copy number
days
35%
30%
25%
20%
15%
10%
5%
0%
1.E+9
1.E+8
1.E+7
1.E+6
1.E+5
1.E+4
1.E+3
1.E+2
0
2
4
6
8
10
12
14
Bacillus drentensis
Arthrobacter ramosus
Paeniglutamicibacter cryotolerans
Arthrobacter gyeryongensis
16 rRNA copy number

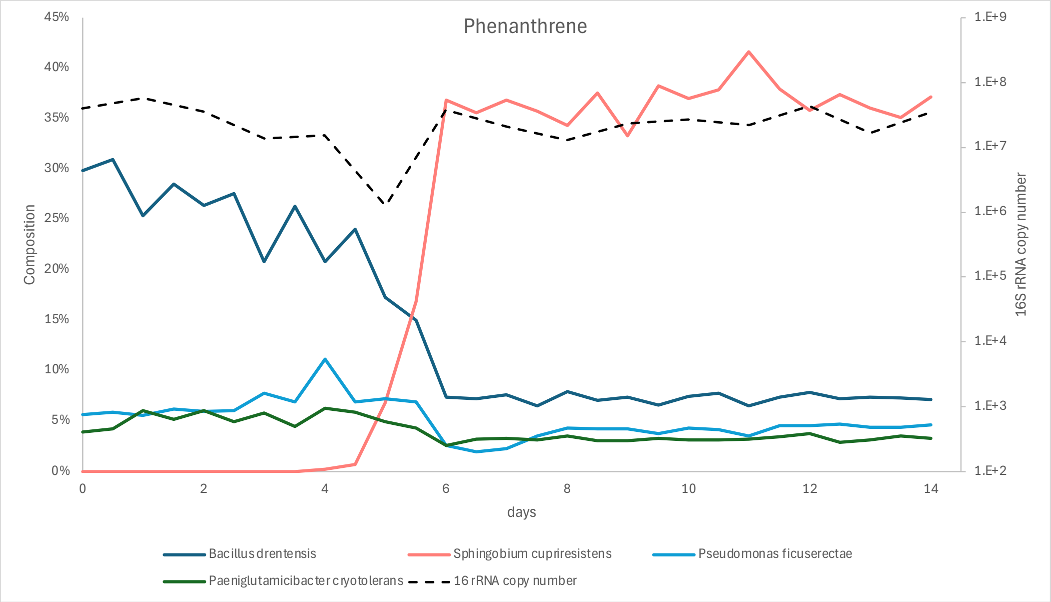
Phenanthrene
Composition
45%
40%
35%
30%
25%
20%
15%
10%
5%
0%
1.E+9
1.E+8
1.E+7
1.E+6
1.E+5
1.E+4
1.E+3
1.E+2
16S rRNA copy number
0
2
4
6
8
10
12
14
days
Bacillus drentensis
Sphingobium cupriresistens
Pseudomonas ficuserectae
Paeniglutamicibacter cryotolerans
16 rRNA copy number

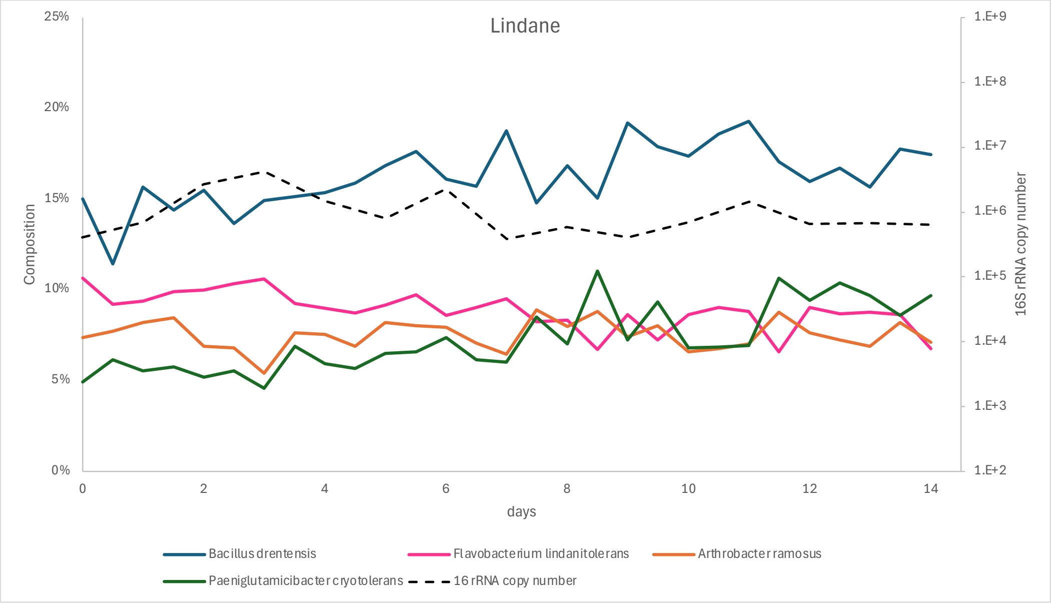
Lindane
Composition
25%
20%
15%
10%
5%
0%
1.E+9
1.E+8
1.E+7
1.E+6
1.E+5
1.E+4
1.E+3
1.E+2
16S rRNA copy number
0
2
4
6
8
10
12
14
days
Bacillus drentensis
Flavobacterium lindanitolerans
Arthrobacter ramosus
Paeniglutamicibacter cryotolerans
16 rRNA copy number

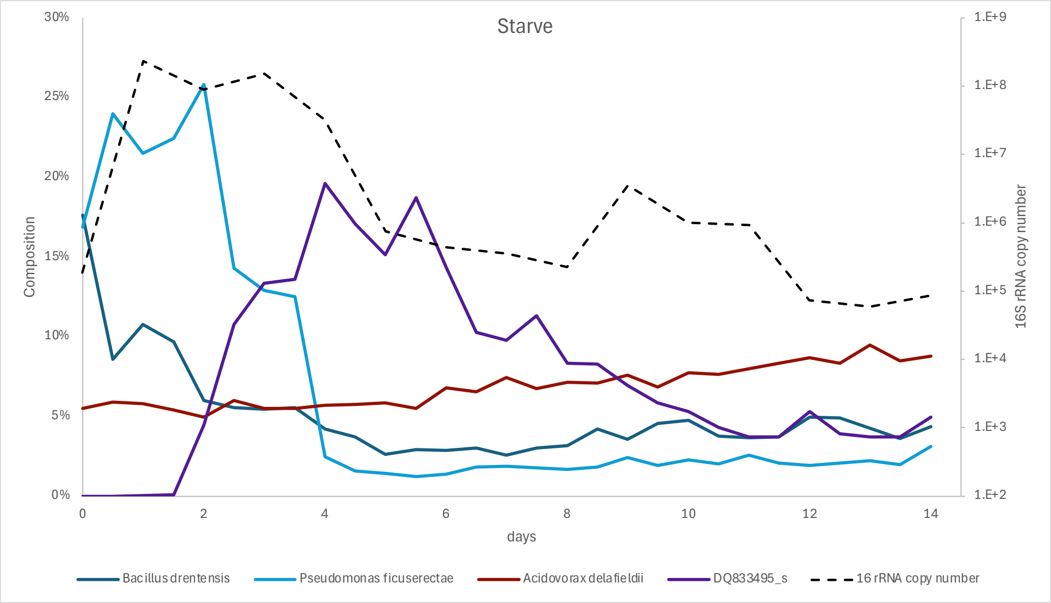
Starve
Composition
30%
25%
20%
15%
10%
5%
0%
1.E+9
1.E+8
1.E+7
1.E+6
1.E+5
1.E+4
1.E+3
1.E+2
16S rRNA copy number
0
2
4
6
8
10
12
14
days
Bacillus drentensis
Pseudomonas ficuserectae
Acidovorax delafieldii
DQ833495_s
16 rRNA copy number

Figure S5: Temporal changes in the relative abundance of the most dynamic bacterial genera across the six experimental conditions. The dashed line in each panel represents the total bacterial population size (16S rRNA gene copies/mL, right y-axis), providing context for community growth or decline. These graphs provide the complete dataset supporting the successional patterns discussed in the main text (Section 3.3.2) and illustrated in Figure 1. ***(A) Glucose:*** *Demonstrates a classic r-strategist bloom-and-bust pattern. Fast-growing Neobacillus drentensis dominates early but declines as glucose is depleted, followed by the rise of secondary consumers like Pseudomonas and Mucilaginibacter that likely feed on necromass or metabolic byproducts.* ***(B) Succinate:*** *Shows more complex competitive dynamics. Neobacillus drentensis remains a key player, but its dominance fluctuates, indicating sustained co-existence and competition with other genera like Arthrobacter and Ensifer.* ***(C) Naphthalene:*** *After an initial lag phase of ~4 days, the community shifts. Paeniglutamicibacter and Arthrobacter become dominant as naphthalene decreases.* ***(D) Phenanthrene:*** *Exhibits the most dramatic successional event. Following a lag phase, the specialist degrader Sphingobium cupriresistens rapidly outcompetes the initial community, rising to >20% relative abundance and demonstrating powerful selection for a specific metabolic capability.* ***(E) γ-HCH:*** *Under the stress of a toxic, recalcitrant substrate, the initial dominant taxa decline. The community becomes enriched for tolerant species, notably Flavobacterium lindanitolerans, which likely survives by scavenging necromass from less tolerant bacteria.* ***(F) Starve:*** *In the absence of an external carbon source, the total population steadily declines. The community composition shifts towards resilient K-strategists (e.g., Acidovorax) that can persist under severe nutrient limitation while the initial bloomers fade.*

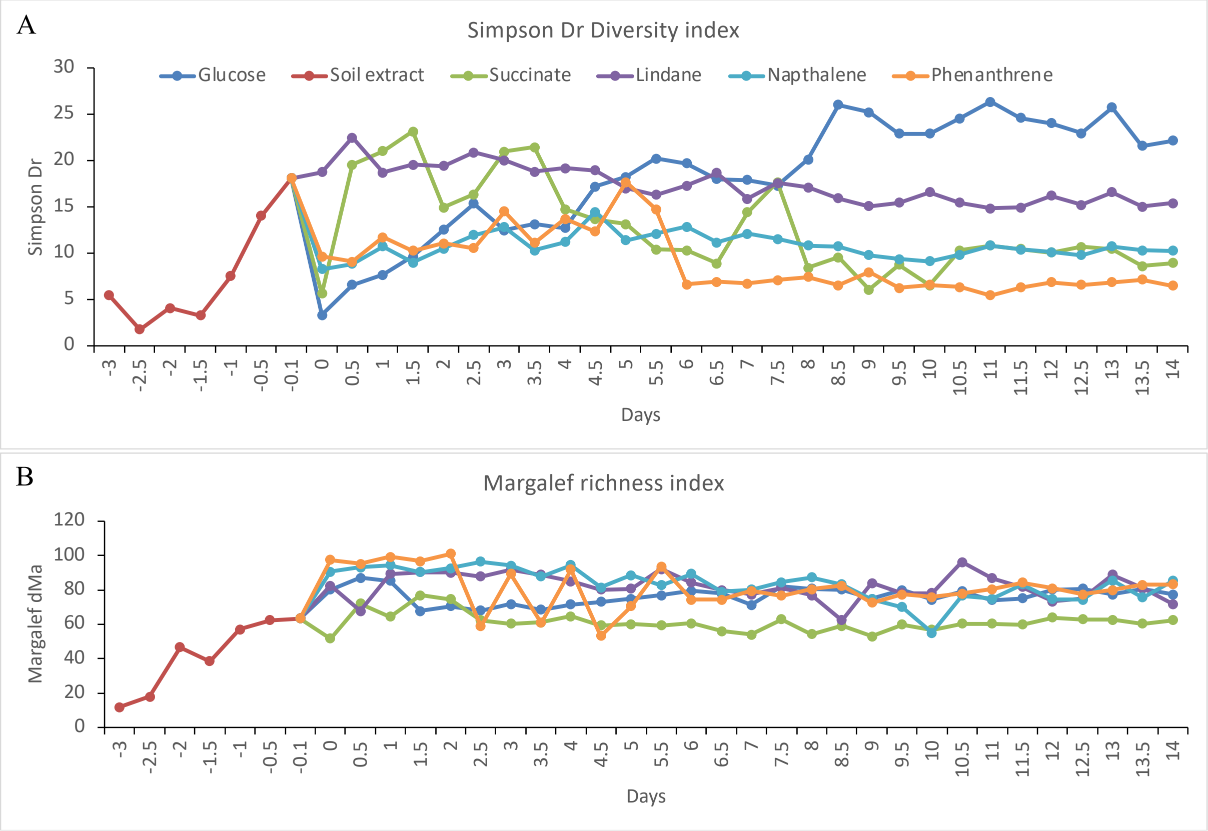

Figure S6: Community diversity and richness dynamics. ***(A) Simpson's Index of Diversity (Dr)*** *and* ***(B) Margalef's Richness Index (dMa)*** *were calculated for all treatments over time. The initial soil extract community (day 0) shows high diversity. Diversity and richness patterns fluctuate based on the carbon source, with a general trend of decreasing diversity during the initial bloom of fast-growing specialists, followed by a stabilization or slight recovery. Each point is the average of three biological triplicates.*

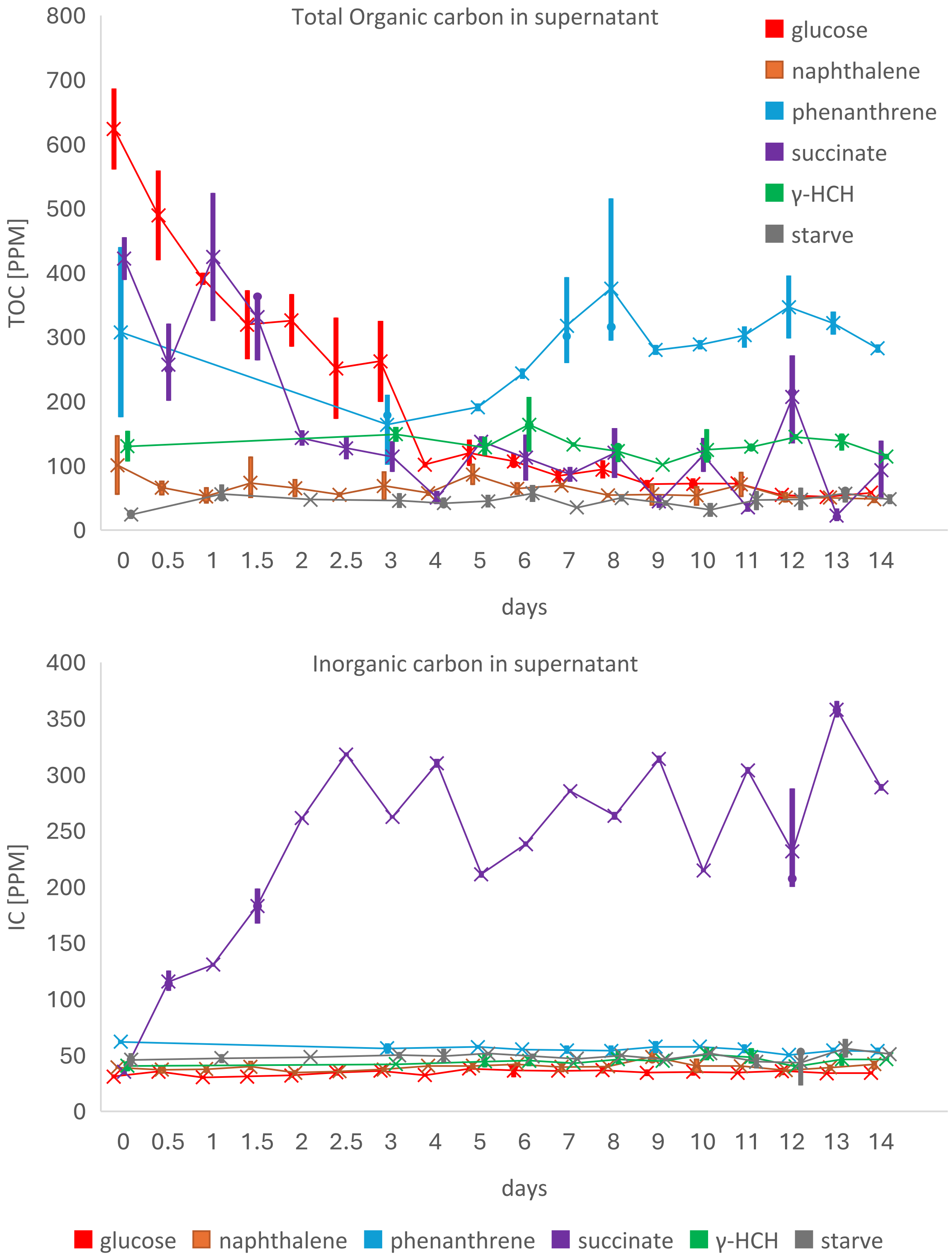

Figure S7: Analysis of Soluble Carbon Pools Reveals Substrate Mineralization and Byproduct Formation. This figure shows the dynamics of ***(A)*** *Total Organic Carbon (TOC) and* ***(B)****Inorganic Carbon (IC) in the culture supernatant, providing mechanistic insights into substrate consumption and its effect on the environment. Data points represent the mean of three biological triplicates, and error bars indicate the standard deviation.* ***(A) Total Organic Carbon (TOC):*** *This panel confirms the rapid consumption of the soluble substrates, glucose and succinate, consistent with the depletion data in Figure 1. The low TOC levels in naphthalene, phenanthrene, and γ-HCH cultures reflect their poor water solubility. Notably, the gradual rise in soluble TOC for the phenanthrene treatment provides direct evidence for the biotransformation of the insoluble parent compound into soluble metabolic byproducts.* ***(B) Inorganic Carbon (IC):*** *This panel tracks substrate mineralization. A sharp and significant increase in IC to ~250 mg/L is observed exclusively in the succinate cultures within the first two days. In contrast, all other treatments maintained a stable baseline IC level of approximately 50 mg/L. This rapid accumulation of inorganic carbon suggests intense mineralization of succinate, which likely induced a secondary environmental stress (e.g., a decrease in pH), explaining why it supported a lower final bacterial population density than glucose (Figure 2).*

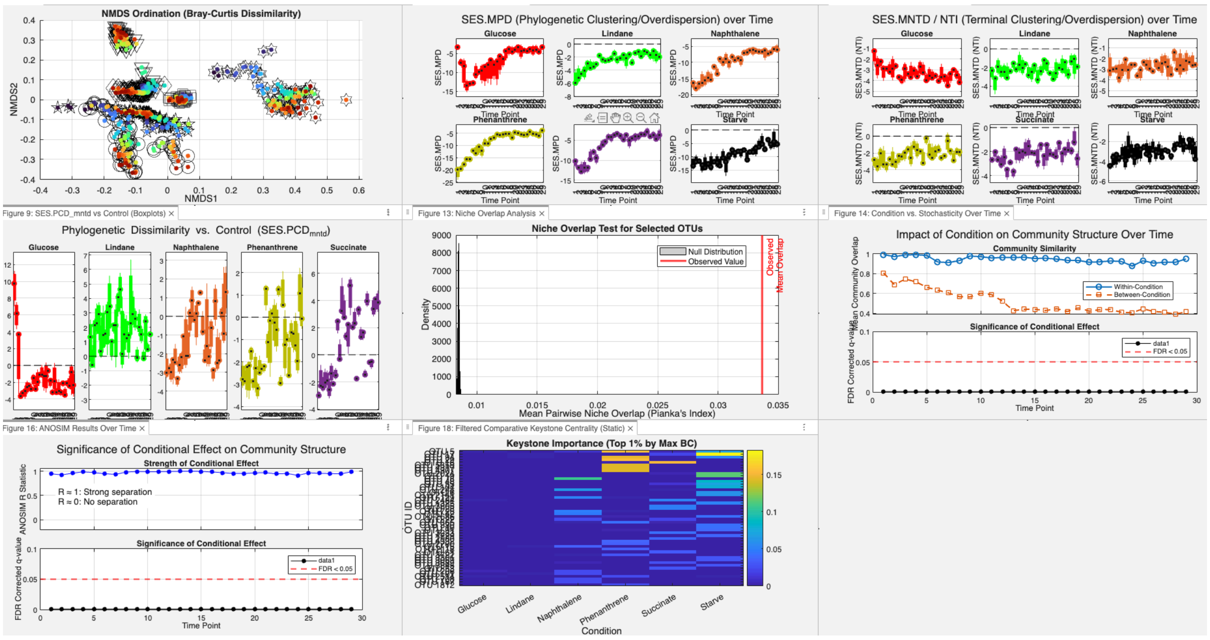

Figure S8: Comprehensive Analysis of Microbial Community Dynamics and Assembly Mechanisms. This figure presents a suite of analyses performed on the microbial community data using the whole dataset (5462 OTUs). ***(a) Non-metric Multidimensional Scaling (nMDS)*** *ordination based on Bray-Curtis dissimilarity, showing distinct clustering of microbial communities according to the experimental condition (carbon source).* ***(b) Standardized Effect Size of Mean Pairwise Distance (SES.MPD)*** *over time, indicating significant phylogenetic clustering (values < 0) in most conditions, a signature of environmental filtering.* ***(c) Standardized Effect Size of Mean Nearest Taxon Distance (SES.MNTD or NTI****) over time, confirming terminal phylogenetic clustering and reinforcing the environmental filtering hypothesis.* ***(d) Boxplots of phylogenetic dissimilarity (SES.PCD)*** *for each condition relative to starve control, illustrating the varying degrees of phylogenetic divergence driven by different substrates.* ***(e) Niche overlap test for selected OTU****s. The observed mean pairwise niche overlap (red line) is significantly higher than the null distribution, indicating that dominant organisms share similar niches, consistent with environmental filtering rather than competitive exclusion****. (f) Impact of experimental condition on community structure****. The top panel shows that community similarity is consistently higher within a given condition (blue line) than between different conditions (orange line), and the bottom panel confirms this effect is statistically significant (FDR < 0.05) at all time points****. (g) ANOSIM results over time****. The R-statistic (top panel) is consistently high, indicating strong separation between community structures based on condition, which is confirmed to be significant by the FDR-corrected q-values (bottom panel).* ***(h) Heatmap of keystone importance*** *(top 1% by max betweenness centrality) for the most influential OTUs across the different experimental conditions, highlighting that each substrate selects for a distinct set of structurally important taxa.*

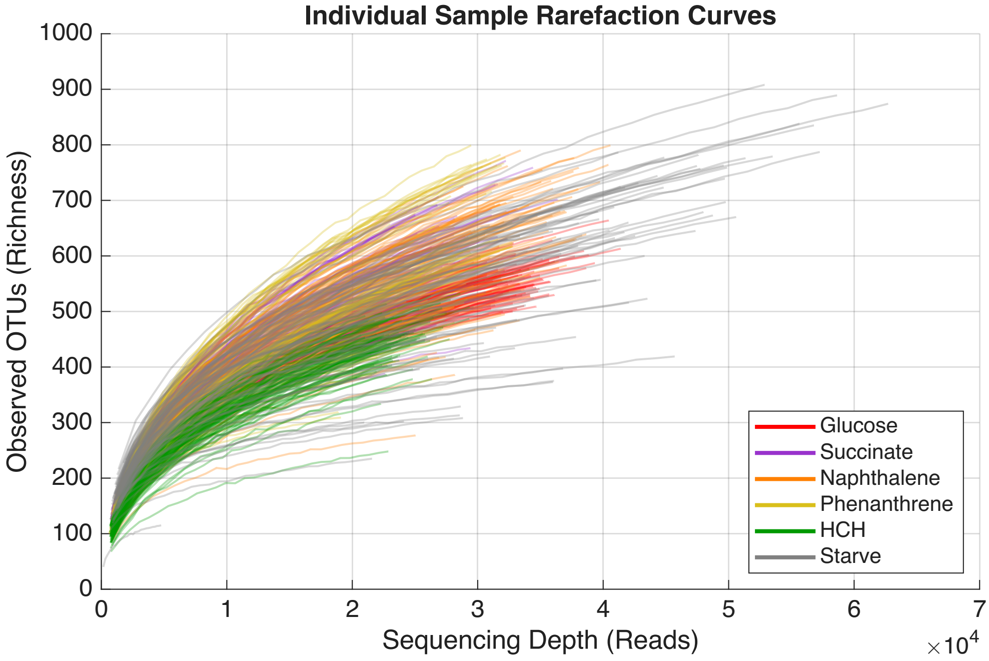

Figure S9: Individual sample rarefaction curves of bacterial communities under different carbon source conditions. The plot displays the accumulation of observed Operational Taxonomic Units (OTUs, clustered at 97% similarity) as a function of sequencing depth (number of reads) for each individual sample. Data are separated by carbon source treatment (Glucose, Succinate, Naphthalene, Phenanthrene, γ-HCH, and Starved control). The curves exhibit an asymptotic trend, generally plateauing between 20,000 and 60,000 reads. This indicates that the per-sample sequencing depth was sufficient to capture the majority of bacterial diversity within the microcosms. Consistent with the main text, the Starved control samples (grey) generally exhibit higher accumulated richness, likely reflecting the persistence of a diverse "seed bank" (rare biosphere) in the absence of competitive exclusion by fast-growing specialists. In contrast, the γ-HCH condition (green) consistently shows lower richness, consistent with the selective pressure of toxicity.

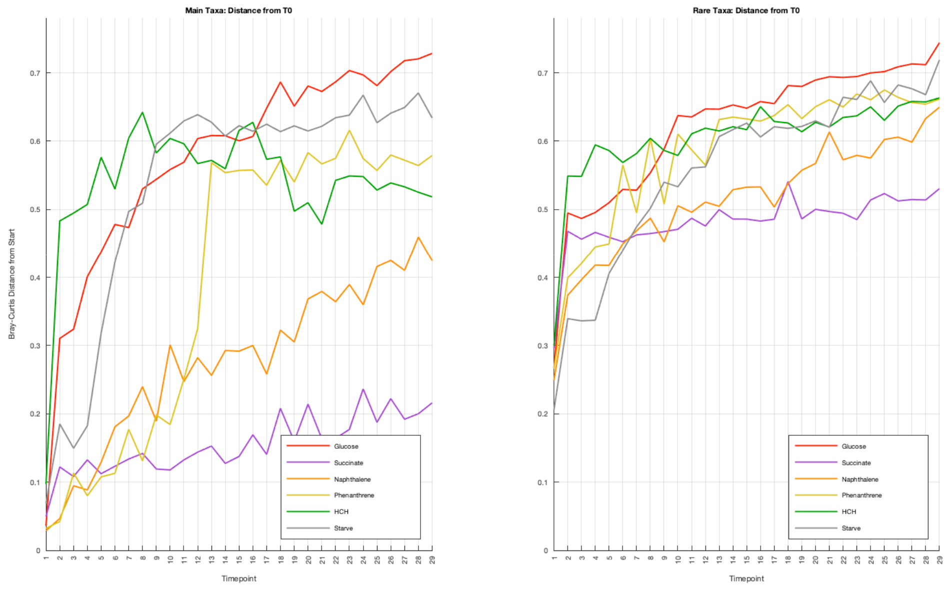

Figure S10: Decoupled temporal divergence of Main and Rare bacterial subcommunities. The plots track the compositional turnover of the community relative to the initial inoculum (T0) over 29 time points (14 days), calculated as the Bray-Curtis dissimilarity from T0. ***(Left) Main Taxa*** *(OTUs >1% mean relative abundance): The abundant subcommunity shows substrate-dependent trajectories. While Glucose (red) and Phenanthrene (yellow) drive rapid divergence from the initial state (with Phenanthrene showing a sharp shift around time point 12, coinciding with the onset of degradation), the Succinate (purple) community remains relatively similar to the starting soil extract community.* ***(Right) Rare Taxa*** *(OTUs <0.1% mean relative abundance): In contrast to the Main taxa, the rare biosphere exhibits a rapid and high degree of divergence from the inoculum across all conditions, regardless of the carbon source. Notably, even in the Succinate condition (purple), where the Main taxa were stable, the Rare taxa shifted significantly, visually confirming that the rare and abundant subcommunities are governed by distinct successional dynamics.*

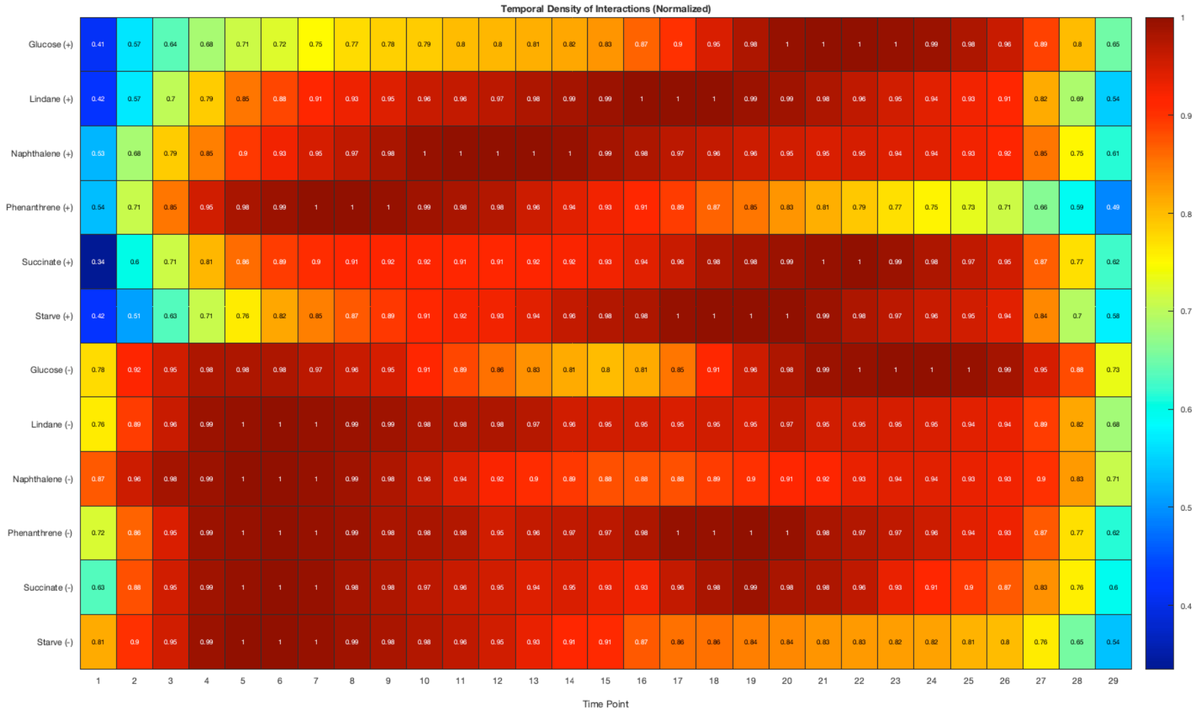

Figure S11: Normalized temporal density of interactions (eLSA). The heatmap displays the temporal evolution of significant pairwise associations inferred by Extended Local Similarity Analysis (eLSA) across the 29 time points. The y-axis separates interactions by sign (Positive (+) or Negative (-)) and condition (Note: "Lindane" in the figure refers to the γ-HCH condition). The color gradient (blue to dark red) represents the normalized density of significant interactions active at each time point. Negative interactions (-) (bottom rows) remain consistently high (orange/red) throughout the incubation for most conditions, particularly Glucose, reflecting the "Global" nature of competitive exclusion where dominant taxa suppress others. Positive interactions (+) (top rows) exhibit condition-dependent dynamics. Consistent with the Stress Gradient Hypothesis described in the text, the γ-HCH (Lindane) and Starve conditions show a rapid onset and high density of positive interactions (facilitation) compared to the nutrient-rich Glucose condition. This supports the conclusion that the community shifts toward cooperative strategies (e.g., cross-feeding or detoxification) under environmental stress, whereas resource abundance favors competition.

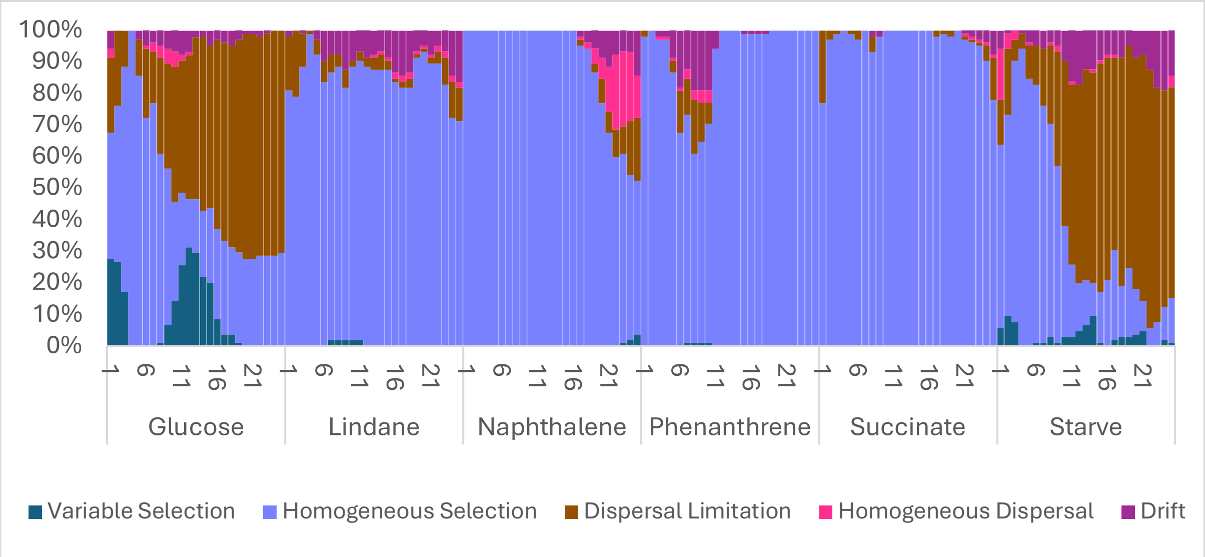

Figure S12: Temporal dynamics of ecological assembly processes within replicates. A stacked bar chart illustrates the changing relative contribution of ecological processes over time for each condition (calculated using a 5-point rolling window). Colors correspond to: Variable Selection (Dark Teal), Homogeneous Selection (Light Blue), Dispersal Limitation (Brown), Homogenizing Dispersal (Pink), and Ecological Drift (Purple). The data reveals a clear link between active metabolism and deterministic assembly: (1) Conditions with continuous pressure (e.g., Succinate, Naphthalene, Lindane) are dominated by Homogeneous Selection, indicating that metabolic constraints maintain high reproducibility between replicates. (2) In Glucose, the depletion of the substrate (approx. midway through incubation) coincides with a shift from Homogeneous Selection to stochastic Dispersal Limitation, as the selective pressure is removed. (3) Conversely, Phenanthrene initially exhibits stochastic assembly (Dispersal Limitation and Drift) during the metabolic lag phase, shifting to deterministic Homogeneous Selection only after degradation actively begins (approx. day 6). (4) The Starved control consistently shows high levels of Dispersal Limitation, confirming that in the absence of a strong nutrient filter, community assembly is driven largely by stochastic historical contingencies rather than deterministic selection.

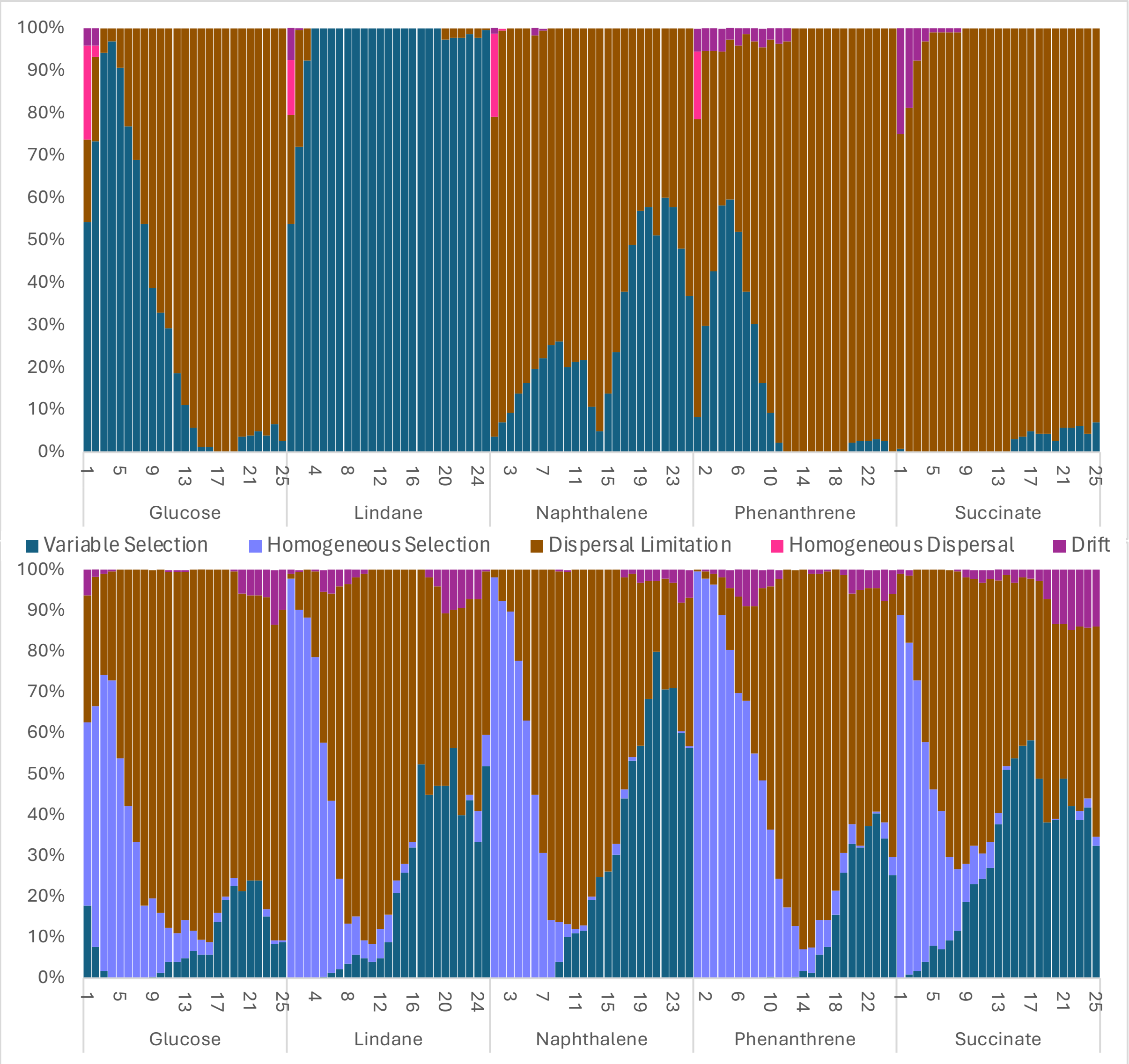

Figure S13: Decoupled temporal dynamics of ecological assembly processes for Main vs. Rare subcommunities. Stacked bar charts illustrate the changing relative contribution of ecological processes over time, calculated by comparing each carbon source treatment against the Starved control (using a 5-point rolling window). Colors correspond to: Variable Selection (Dark Teal), Homogeneous Selection (Light Blue), Dispersal Limitation (Brown), Homogenizing Dispersal (Pink), and Ecological Drift (Purple). ***(Top) Main Bacteria (>1%):*** *Exhibit an immediate deterministic response to substrate availability. Variable Selection dominates early phases, particularly in Glucose and Lindane, acting as the primary filter.* ***(Bottom) Rare Bacteria (<0.1%):*** *Exhibit a latent deterministic response. Early stages are dominated by Homogeneous Selection and Dispersal Limitation, consistent with a "seed bank" dynamic shaped by general survival constraints. However, a delayed surge in Variable Selection occurs in the later stages of incubation, confirming that the rare biosphere responds deterministically as secondary responders to downstream environmental changes rather than the initial substrate.*
